# Morphometrics of the preserved post-surgical hemisphere in pediatric drug-resistant epilepsy and implications for post-operative cognition

**DOI:** 10.1162/IMAG.a.1194

**Published:** 2026-04-06

**Authors:** Michael C. Granovetter, Anne Margarette S. Maallo, Christina Patterson, Daniel Glen, Marlene Behrmann

**Affiliations:** Department of Psychology and Neuroscience Institute, Carnegie Mellon University, Pittsburgh, PA, United States; School of Medicine, University of Pittsburgh, Pittsburgh, PA, United States; Departments of Pediatrics and Neurology, New York University, New York, NY, United States; Department of Pediatrics, University of Pittsburgh, Pittsburgh, PA, United States; Scientific and Statistical Computing Core, National Institute of Mental Health, Bethesda, MD, United States; Department of Ophthalmology, University of Pittsburgh, Pittsburgh, PA, United States

**Keywords:** MRI, structure, thickness, volume, epilepsy surgery, pediatrics, development, RRID:SCR_023356

## Abstract

Characterization of the structural integrity of cortex in adults who have undergone resection for epilepsy treatment has revealed persistent or even accelerated cortical atrophy in some cases, but, in other cases, the converse is evident, and atrophy can decelerate or even be reversed. Whether this variability applies to a pediatric population, for whom postoperative plasticity may be greater than in adulthood, remains to be determined. Furthermore, understanding the morphometrics of this patient population is important, as cognitive gains have been associated with the anatomical status of the preserved cortex post-resection. Here, we used high-resolution structural T1 magnetic resonance imaging data to compare the (1) gross anatomy, (2) cortical thickness, volume, and surface area for 34 cortical regions, and (3) volume for nine subcortical regions of 32 pediatric post-surgical cases and 51 healthy controls. We only analyze the metrics from the preserved hemisphere. Relative to controls, patients with either a preserved right hemisphere (RH) or left hemisphere (LH) had significantly lower total white matter volume and larger lateral ventricle size, as well as a reduction of the volume of select subcortical structures. However, relative to controls, only patients with a preserved RH had significantly lower total gray matter volume and lower thickness, volume, and surface area in multiple cortical regions, primarily in the frontal and temporal cortex. The differences in the preserved RH cortex of LH resection patients may relate to transfer of language function from the affected LH. Our findings lay the foundation for future studies probing associations of the morphometric differences in pediatric epilepsy surgery patients with neuropsychological outcomes.

## 1 Introduction

Epilepsy is a complex, progressive neurological disorder characterized by disruption of the normal balance of excitation and inhibition and sudden spikes of electrical discharge, all of which may result in neuronal impairment, axonal damage, and altered neural circuitry (Rossini et al., 2021). Pharmacologic treatment is often successful for seizure management, but, for ~7–20% of pediatric and ~30–40% of adult patients with drug-resistant epilepsy (DRE) (Tang et al., 2017; Xue-Ping et al., 2019), seizure control can be achieved by surgical resection of epileptogenic cortex. Post-surgical seizure freedom occurs in ~86% and ~81% of patients at 1 and 2 years follow-up, respectively (Weil et al., 2020) (see also Jobst & Cascino, 2015), and significant gains in several areas of cognition are reported in many individuals (Helmstaedter et al., 2020).

Because the post-surgical neuropsychological status relies largely on the post-surgical structural integrity of non-resected cortex, understanding the morphometrics of this residual tissue is important. Pre-surgical persistent and possibly accelerated reduction in cortical thickness (CxT)—a proxy for structural integrity of the brain (Federico & Wiebe, 2020)—has been identified beyond the defined epileptogenic zone/lobe and is evident even in the preserved hemisphere (Caciagli et al., 2017; Fonseca et al., 2023; Galovic et al., 2019; Grinenko et al., 2018), although this might be limited to a subset of regions. This reduction in gray matter (GM) appears to be ubiquitous across different epilepsy syndromes (Whelan et al., 2018). In contrast to these findings of ongoing structural compromise, post-surgical halting of progressive cortical atrophy has also been reported (Galovic et al., 2019, 2020; Lopez et al., 2022; Park et al., 2022) and, dramatically, in some patients with temporal lobe epilepsy, the pre-surgical reduction in CxT can even be reversed to normal rates (Galovic et al., 2019) (for commentaries, see McDonald, 2021 and Federico & Wiebe, 2020). Importantly, the extent of reversal is correlated with better seizure control (Federico & Wiebe, 2020; Galovic et al., 2019, 2020), as well as positive cognitive outcomes (Skirrow et al., 2011). There is also reversal of not only reduced CxT, but also reduced cortical volume (CV), and this recovery, too, is positively associated with cognitive improvement post-resection (Zhao et al., 2021). Moreover, in an encompassing recent review of patients with temporal lobe epilepsy surgery, three possible interpretations of post-surgical changes are enumerated: alterations that lead to damage and degeneration, recovery, or reorganization (Sainburg et al., 2025). Which of the three outcomes one should expect and for which region remains to be determined.

One key limitation of understanding the morphometrics in the postoperative period is that most existing studies focus primarily on adults. One of the few such studies found increased GM volume in the temporal cortex and decreased volume in the limbic cortex—as well as decreased CxT in several regions—of the preserved hemisphere of pediatric hemispheric surgery patients (Zhao et al., 2025). An understanding of morphometric differences in children post-resection is of high priority. This is especially important given the greater potential for plasticity post-surgically in children than in adults, (Fandakova & Hartley, 2020; Hubener & Bonhoeffer, 2014) and extrapolating from the findings from studies conducted with adults may be misleading. Studies of the anatomical status of post-surgical cortex are also sorely needed given the growing consensus that surgery is underutilized and should be considered a first-line intervention for DRE, especially in childhood (Consales et al., 2021; Cross et al., 2022; Vakharia et al., 2018).

Additionally, cognitive outcomes may rely on the extent of pre- to post-surgical anatomical changes. For example, in one study of children who had undergone temporal lobe surgery, better verbal and semantic memory were associated with extent of residual hippocampal volume and temporal lobe integrity in left surgical patients (however, no other regions or metrics were measured in this investigation) (Skirrow et al., 2015). Furthermore, Zhao et al. (2025) examined GM structure pre- and post-hemispherotomy using voxel- and surface-based morphometry, with a specific focus on correlations with motor function after surgery. While normalization of prefrontal and cingulate cortical morphometry was associated with better motor function, there was GM volume loss of the temporal lobe that was associated with greater functional deficits. While both studies are informative, the first included only patients aged 16 years and older, precluding the opportunity to evaluate structural changes at earlier ages, and the second focused specifically on GM cortical structure.

Here, we compared the post-surgical morphometry of the preserved hemisphere in 32 children with DRE—ranging in age from 7–22 years—and 51 age-matched typically developing controls on (1) gross total volumes of the lateral ventricle (LV), GM, and white matter (WM); (2) CxT (the average thickness or distance between the white and pial surfaces), CV, and cortical surface area (CSA), separately for 34 automatically-segmented cortical parcels; and, last, (3) volume of nine subcortical structures. CxT, CV, and CSA, in particular, are metrics that are commonly adopted in studying brain structure (Azzony et al., 2023) and have distinct genetic profiles, lifespan trajectories, links with neuropsychological factors, and disease associations (Frangou et al., 2022; Lyall et al., 2015; Wierenga et al., 2014). These metrics are also differentially associated with cognitive development and neurodevelopmental disorders (Fjell et al., 2015; Williams et al., 2023; Winkler et al., 2010): whereas CxT development—which has been associated with intelligence (Schnack et al., 2015)—follows a linear decrease with age (Sanders et al., 2022), CSA and CV follow a curvilinear trajectory, with CV peaking earlier than CSA (Wierenga et al., 2014). The information that we can glean from these different measures, thus, offers unique and complementary insights. Moreover, although post-surgical reduction in CxT affects both hemispheres equally in adults (Galovic et al., 2020), here, we were interested in whether any post-surgical structural differences depended on the preserved hemisphere being left or right following childhood resection.

We focus our investigation and analyses solely on the preserved hemisphere, as any residual tissue in the hemisphere ipsilesional to surgery may have structural abnormalities either directly related to the surgery or because of ongoing epileptogenic activity. Unlike prior studies, we include patients who have had a range of pediatric epilepsy surgeries, and we examine total volumes, multiple individual morphometrics of parcellations, and subcortical volumes, all in a single sample. We employed general linear modeling (GLM) to compare gross anatomy, cortical morphometry, and subcortical morphometry of patients versus controls and, separately, patients’ preserved left hemisphere (LH) vs. patients’ preserved right hemisphere (RH). Because univariate analysis may be plagued by covariance between morphological variables (Wang et al., 2021), we also exploited multivariate analyses to identify models that include a combination of structural parameters. We use these latter models to predict group assignment (patient vs. control) and, in the patients, any ongoing seizure burden, using the International League Against Epilepsy (ILAE) outcome classification schema (Fisher et al., 2017).

## 2 Methods

### 2.1 Participants

We recruited 32 pediatric patients with DRE and subsequent cortical resection or ablation for this study, either referred to us by Dr. Christina Patterson, Director, Epilepsy and EEG Services, the University of Pittsburgh Children’s Hospital or recruited from the Pediatric Epilepsy Surgery Alliance advocacy group (https://epilepsysurgeryalliance.org/). Participants needed to be able to undergo magnetic resonance imaging (MRI) without sedation. Additionally, no patients had hydrocephalus significant enough necessitating a shunt. The group included 13 patients with LH surgery and a preserved RH (median age/median absolute deviation of age: 15.7/1.7 years; 6 females, 7 males) and 19 patients with RH surgery and a preserved LH (median age/median absolute deviation of age: 15.4/3.7 years; 11 females, 8 males). The patients’ demographic information and medical history are detailed in Table 1. This includes age at testing, age at surgery, age at seizure onset, and time from surgery to testing (subtracting age at seizure onset from age at testing). Note that we use “RH patient” to refer to patients with left-sided resections but a preserved RH, as it is the RH from which we derive the morphometric measures, and the same holds true for “LH patient” in reference to the preserved LH of these patients. Any reference to “LH resection” or “RH resection”, however, is in explicit reference to resection of the LH or RH, respectively.

**Table 1.** Patient information.

Age at test (yr)	Gender	Age at surgery (yr)	Seizure onset age (yr)	Time from surgery to testing (yr)	ILAE Scale (Binned)	Surgery
	Left Surgery Patients/Preserved Right (RH patients)					
11.5	Male	1	0	11	Low	Hemispherectomy
12.5	Male	0	-	13	Low	Evacuation of temporal hematoma
13.9	Female	13	6	1	Low	Occipital and parietal lobectomy
14.7	Male	13	12	2	Low	Temporal lobectomy with preservation of medial structures & gross total resection of enhancing medial temporal lobe tumor
15.1	Female	14	8	1	Low	Frontal lobe resection
15.6	Female	13	6	3	Low	Functional hemispherectomy
15.7	Male	15	7	1	Low	Frontal lesionectomy with corticectomy
16.1	Male	10	0	6	High	Temporal lobectomy with amygdalohippocampectomy
16.7	Female	16	13	1	High	Robot-assisted stereotactic laser ablation of mesial temporal lobe including amygdala and hippocampus (x2)
16.8	Male	4	0	13	Low	Functional hemispherectomy
19.8	Female	4	1	16	Low	Hemidecortication
20.5	Male	18	13	3	Low	Resection of T1 gyrus & Heschl gyrus seizure onset zone
22.2	Female	17	11	5	Low	Amygdalohippocampectomy, anterior temporal lobectomy, & resection of mesial temporal lobe tumor
	Right Surgery Patients/Preserved Left (LH patients)					
7.8	Female	3	2	5	Low	Hemispherotomy
7.8	Male	6	4	2	Low	Occipital lobectomy, posterior temporal lobectomy, & resection of medial inferior temporal lobe tumor
10.9	Female	10	4	1	High	Robot-assisted stereotactic laser thermal ablation of frontal seizure onset zone
11.3	Male	10	6	1	High	Robot-assisted stereotactic laser thermal ablation of amygdala & hippocampus
11.6	Female	4	4	8	High	Temporal lobe lobectomy with amygdalohippocampectomy
13.1	Female	12	10	1	High	Robot-assisted stereotactic laser thermal ablation of operculum
14.4	Female	13	3	1	High	Robot-assisted stereotactic laser thermal ablation of amygdalar seizure onset zone
14.8	Female	8	0	7	Low	Functional hemispherectomy
15.0	Female	10	9	5	Low	Resection of frontal operculum, frontal pole, & inferior frontal lobe
15.4	Male	15	4	0	High	Robot-assisted stereotactic laser thermal ablation of mesial temporal lesion
16.1	Female	1	0	15	Low	Hemispherotomy
16.6	Male	16	11	1	Low	Robot-assisted stereotactic laser ablation of occipito-temporal seizure onset zone (x2)
17.0	Female	15	13	2	Low	Anterior temporal lobectomy & hippocampectomy
17.4	Female	15	10	2	Low	Resection of parietal lobe, premotor, & supplementary motor area
17.8	Male	17	16	1	Low	Gross total resection of frontal brain tumor
18.3	Male	12	7	6	Low	Parietal lobectomy & posterior temporal lobectomy
18.7	Female	9	0	10	Low	Hemispherectomy
19.4	Male	14	6	5	High	Partial resection of parietooccipital tumor (x2)
20.1	Male	18	10	2	Low	Robot-assisted stereotactic laser thermal ablation of insulo- opercular cortex

Note: We binned classes 1–3 into the “Low” International League Against Epilepsy seizure outcome scale (better outcome), and we binned classes 4–6 into the “High” scale (poorer outcome).

The two patient groups were matched on age at testing (*F*(1,30) = 1.12; *p* = 0.30), gender (*z*(30) = 0.65; *p* = 0.51), age at first surgery (*F*(1,30) = 0.03; *p* = 0.87), seizure-onset age (*F*(1,29) = 0.01; *p* = 0.93), and binned ILAE outcome scores (Scheffer et al., 2017) (*z*(30) = 1.29; *p* = 0.20). There is also no statistically significant difference in the interval between the time of surgery between the preserved RH and preserved LH groups, with a median interval of 3 and 2 years, respectively. Given the rarity of this patient population, we report retrospective analyses on patient scans from 2013 to 2022. We invited most patients for post-surgical scanning only.

The 51 control participants (median age/median deviation of age: 14.8/4.9 years; 24 females, 27 males) did not differ from the patients in age, either in aggregate, (*F*(1,81) = 1.18; *p* = 0.28) or by hemisphere (LH: *F*(1,68) = 0.17; *p* = 0.69 | RH: *F*(1,62) = 1.88; *p* = 0.18), and we also matched controls to patients on gender (aggregate: *z*(81) = 0.54; *p* = 0.59 | LH: *z*(68) = 0.80; *p* = 0.42 | RH: *z*(62) = 0.06; *p* = 0.95). For consistency, we sought to recruit right-handed controls. We could not match patients and controls on handedness, as we did not perform standardized handedness assessments on patients preoperatively, and postoperatively, surgery affected handedness (e.g., a left hemispherectomy would result in right hemiparesis). However, atypical handedness is not more prevalent in left vs. right pediatric focal epilepsy (Sharawat et al., 2021), and handedness is not associated with cortical morphometric differences (Jang et al., 2017; Ocklenburg et al., 2016).

For all analyses, we compared the preserved hemisphere of the patients to the corresponding hemisphere of the 51 controls. For those with a preserved LH, the comparison is with the LH of the controls, and for those with a preserved RH, the comparison is with the RH of the controls. This is necessitated by the fact that intact hemisphere is a within-subject variable for the controls but a between-subject variable for the patients and is, thus, not factorially orthogonal.

### 2.2 Ethics statement

Where possible, participants signed assent or consent, as appropriate, and guardians consented to a protocol reviewed and approved by the Institutional Review Boards of Carnegie Mellon University (CMU) and the University of Pittsburgh (IRBSTUDY2015_00000013).

### 2.3 MRI protocol

We acquired T1-weighted images using an MPRAGE sequence (1 mm isotropic resolution, TE = 1.97 ms, TR = 2,300 ms, total scan time ≅5 minutes). We obtained images from either a Siemens Verio 3T scanner with a 32-channel head coil at CMU (36 controls, 13 patients) or a Siemens Prisma 3T scanner with a 64-channel head coil at the CMU-Pitt Brain Imaging Data Generation & Education Center (RRID:SCR_023356; 15 controls, 19 patients).

### 2.4 Outcome measures

The anatomical images were preprocessed (motion correction, intensity normalization, and skull stripping) and then segmented with the recon-all pipeline of FreeSurfer (v7.1.0) (Reuter et al., 2010; Ségonne et al., 2004; Sled et al., 1998). We manually inspected the output. As the recon-all pipeline does not generally result in accurate segmentation of the brains of hemispheric surgery patients, for these large resection patients, the intact hemisphere was mirrored using affine and non-linear transformations (lesion_align program in AFNI) previously shown to be robust to anatomical aberrations (Glen et al., 2021; Granovetter et al., 2024; Maallo et al., 2020). This permitted the automatic segmentation of both the true as well as mirrored hemispheres, and only the data from the actual non-resected hemisphere were analyzed.

Morphometric measures were derived from the preserved hemisphere of the patients and, separately, from each hemisphere of the controls, as follows: (1) gross measures of volume of GM, WM, and LV; (2) cortical morphometry for 34 regions of interest (ROIs), parcellated according to the Desikan-Killiany cortical atlas (Desikan et al., 2006; Fischl, van der Kouwe, et al., 2004); and (3) subcortical structures (Fischl et al., 2002; Fischl, Salat, et al., 2004) of nine regions, with the volume of each structure normalized to the total hemisphere volume (e.g., percentage of volume of each structure relative to the sum of GM, WM, and LV volumes). For the cortical morphometry, for each participant, we calculated CxT in mm, which we did not normalize, as per standard guidelines (Westman et al., 2013); CSA in mm^2^ by taking the area of each region and dividing it by the mean of all of the regions in that hemisphere only; and CV in mm^3^ normalized by total hemisphere volume (Fischl & Dale, 2000; Han et al., 2006; Reuter et al., 2012).

### 2.5 Statistical analysis

Data were analyzed in R (TeamCore, 2020) (for packages utilized, see Supplementary Table S1) and SPSS 29.0.1.0. To harmonize the data across the two MRI machines, ComBat (Fortin et al., 2018) was used to model the data as a linear combination of group, age, gender, and scanner, with the assumption that scanner effects have both additive and multiplicative factors (Fortin et al., 2018). Furthermore, for each measure and ROI, data were winsorized so as not to lose any data: we replaced values above the 95th and below the 5th percentile of the distribution with an approximation of the corresponding percentile values (separately for controls and patients, as well as by hemisphere).

Considering the relatively small sample sizes, for each dependent measure, we implemented permutation testing by randomly shuffling the group label, and we fit a GLM with group as the primary predictor of interest and age and gender as covariates. This was repeated 1,000 times to create a distribution of the β-coefficients for the effect of group. A *p*-value was then calculated as the percentage of occurrences in which the absolute value of the β-coefficient from the simulated distribution exceeded the absolute value of the true β-coefficient. Significance was ascertained at an α-criterion of 0.05, and, per measure, the Benjamini-Hochberg correction (Benjamini & Yekutieli, 2001) was applied to *p*-values across the total number of ROIs. In addition, for further adjudication of the findings, Bayes factor (BF) was computed by comparing the model with the group term to a null model without the group term (Lee & Wagenmakers, 2013; Wagenmakers, 2007). This was especially important to examine null results and to obtain further evidence to inform the absence of significant effects. BFs below 0.33 and above 3 were interpreted as evidence for the alternative hypothesis and for the null hypothesis, respectively.

Thereafter, to elucidate which measures, alone or in combination, predicted group membership for patients vs. controls and, then, just between patient groups, we conducted forward binary logistic regression analyses. Such a procedure yields those variables that account for significant variance, over and above any significant variable/s already included in the model. Last, we predicted ILAE outcome score (high vs. low) for the patients using a similar multivariate approach. The *r^2^* value of the regression models is provided using the well-established Nagelkerke measure (a modification of the Cox and Snell R Square, which is derived from the likelihood ratio test statistic (Nagelkerke, 1991)). The *r^2^* value reflects the normalized statistic (scaled between 0 and 1) for the goodness-of-fit of the logistic model, making interpretation of outcome easier than typical regression analyses. Instead of obtaining an absolute *p*-value, the Nagelkerke procedure indicates that a value below 0.2 indicates a weak relationship between the predictors and the outcome, a value of 0.2 to 0.4 indicates a moderate relationship, and a value of 0.4 or higher indicates a strong relationship. Because we are only interested in values indicating at least a moderate or a strong relationship, we only report those computed *r^2^* values greater than 0.25, and, for those below 0.25, we simply report the presence of a weak relationship between the predictors and outcome and do not offer any further interpretation.

## 3 Results

For all analyses, we report the statistical comparisons in the following sequence. First, we report the findings of each GLM, separately for each of the patient groups relative to the corresponding hemisphere of all controls. We then describe the findings from comparisons of the patient groups against each other. Note that age and gender are included as covariates in every analysis. Thereafter, for each comparison, we report the results of the binary logistic regression analyses identifying the factors that best predict each group and ILAE assignment.

### 3.1 Gross anatomical morphometrics

**Fig. 1.**
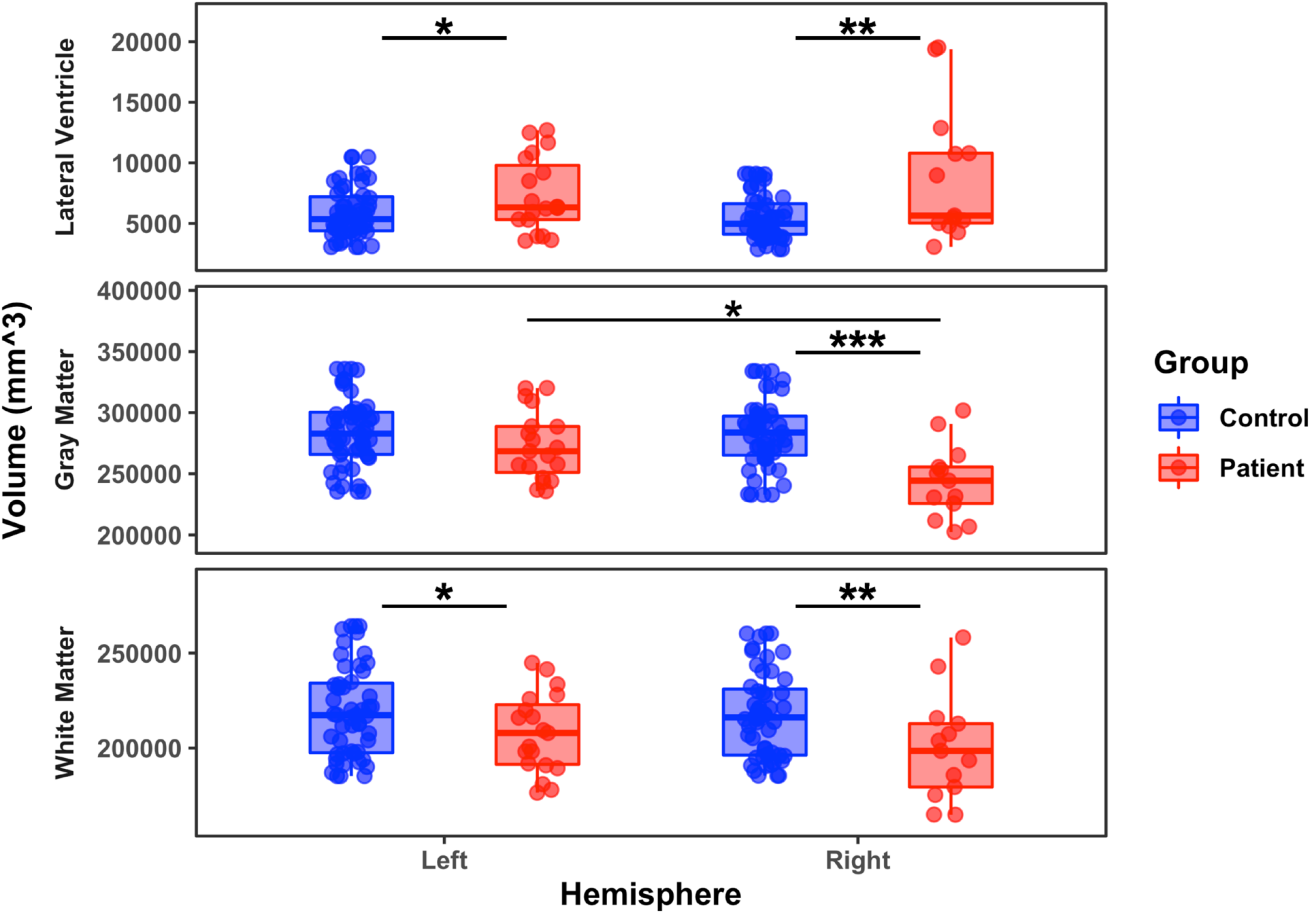
Volume of (top) lateral ventricles, (middle) gray matter, and (bottom) white matter for patients and controls, separately for the preserved left and right hemispheres. *: *p* < 0.05; **: *p* < 0.01; ***: *p* < 0.001. Each dot represents a single participant, and the solid horizontal line in the box indicates the median of the group.

GLM revealed that, relative to the LH of the controls, LH patients had significantly larger LV volume (*p* = 0.02, *BF* = 0.57), significantly smaller WM volume (*p* = 0.05, *BF* = 1.02), and no difference on GM volume (*p* = 0.27, *BF* = 4.37). Relative to the RH of the controls, the RH patient group, like the LH patients, also had significantly larger LV volume (*p* < 0.01, *BF* = 0.01) and significantly smaller WM volume (*p* < 0.01, *BF* = 5.56 × 10^-2^), but they also had significantly smaller GM volume (*p* < 0.001, *BF* = 1.6 × 10^-3^). Across the two patient groups, neither the volume of the LV (*p* = 0.55, *BF* = 4.54) nor the volume of the WM differed (*p* = 0.13, *BF* = 1.39). However, the LH patient group had significantly greater GM volume than the RH patient group (*p* < 0.01, *BF* = 1.98 × 10^-2^). See Figure 1 for these findings. (See Supplementary Table S2 for descriptive statistics.)

To evaluate which of these three dependent measures, LV, GM, and WM, singly or in combination predicted patient/control group membership, we used forward binary logistic regression with *p* < 0.05 for entry criterion. LH patients and LH controls could not be reliably discriminated (model with low *r^2^* indicating a weak relationship between the predictors and outcome). This same analysis with data from RH patients and RH controls yielded a model that included GM and LV, in that order, but not WM, with an *r^2^* of 0.5 (strong relationship). In the direct comparison of the patient groups, GM was the best predictor of preserved hemisphere with *r^2^* = 0.28 (moderate relationship). Last, we examined whether these gross anatomical measures predicted patients’ seizure burden post-surgery and found that no predictor was able to differentiate the ILAE binned outcome scores of the patients.

Patients’ preserved LH differed from controls’ LH on LV and WM, but not GM (although these group differences are not completely well-supported, as we do not replicate them on binary regression analysis). The patients with a preserved RH differed significantly from controls on all three measures of gross anatomy; however, just GM and LV, and not WM, combined into a strongly predictive regression model. Across the two patient groups, GM alone moderately predicted patient group membership, and ILAE outcome could not be predicted by any dependent measure/s.

### 3.2 Cortical regional morphometrics

This section presents the GLM analyses separately for three dependent measures: CxT in mm, SA in mm^2^, and CV in mm^3^ for each of the 34 parcellated ROIs. We then report forward binary regression analyses, as above, separately for the three dependent measures, to determine the model/s that best predict group membership and the binned ILAE outcome scale. Last, we conduct a full multivariate regression in which we include all three dependent variables for each of the 34 regions in the models predicting group membership for each of the LH and RH patient groups against controls’ LH and RH, respectively, and then, predicting membership between the two patient groups.

#### 3.2.1 Cortical thickness

**Fig. 2.**
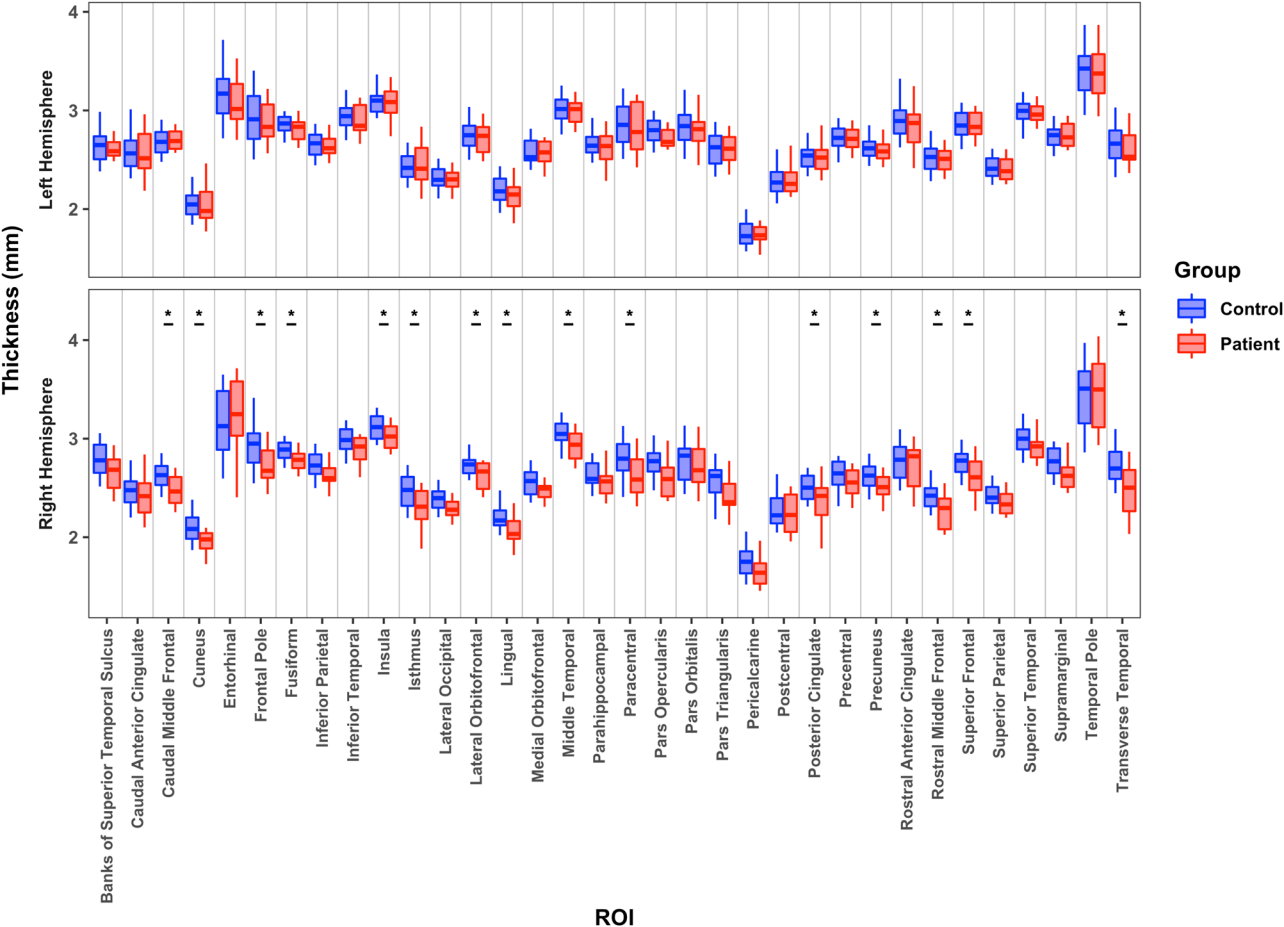
Median cortical thickness for 34 cortical regions for patients’ preserved left hemisphere or right hemisphere relative to controls’ preserved hemisphere. *: *p* < 0.05.

As shown in Figure 2, relative to the LH of controls, the LH patients showed no statistically significant differences in CxT in any of 34 areas. In stark contrast, compared to the RH of controls, the RH patients had significantly lower CxT in 15 of the 34 cortical regions, and there was no region with significantly greater CxT for the patients than controls. Between the patient groups, the RH patients had lower CxT than the LH patients, but this held statistically in only three regions: the caudal middle frontal, rostral middle frontal, and superior frontal regions. (See Supplementary Table S3 for descriptive statistics and Supplementary Table S4 for individual *p* and *BF* values).

A forward regression analysis with CxT indices of all 34 regions of LH patients vs. LH controls resulted in no predictors passing initial criterion for inclusion, indicating that we cannot differentiate patients from controls. A model with a very strong association between the predictors and RH patient vs. RH control group, *r^2^* = 0.8, included the CxT of the caudal middle frontal, frontal pole, lateral occipital, lingual, pars orbitalis, and transverse temporal regions. A model with very strong prediction, *r^2^* = 0.89, which included CxT of entorhinal, rostral middle frontal, and superior temporal regions differentiated LH from RH patients. No viable model was able to separate the patients into high vs. low binned ILAE outcome scores.

#### 3.2.2 Cortical surface area

**Fig. 3.**
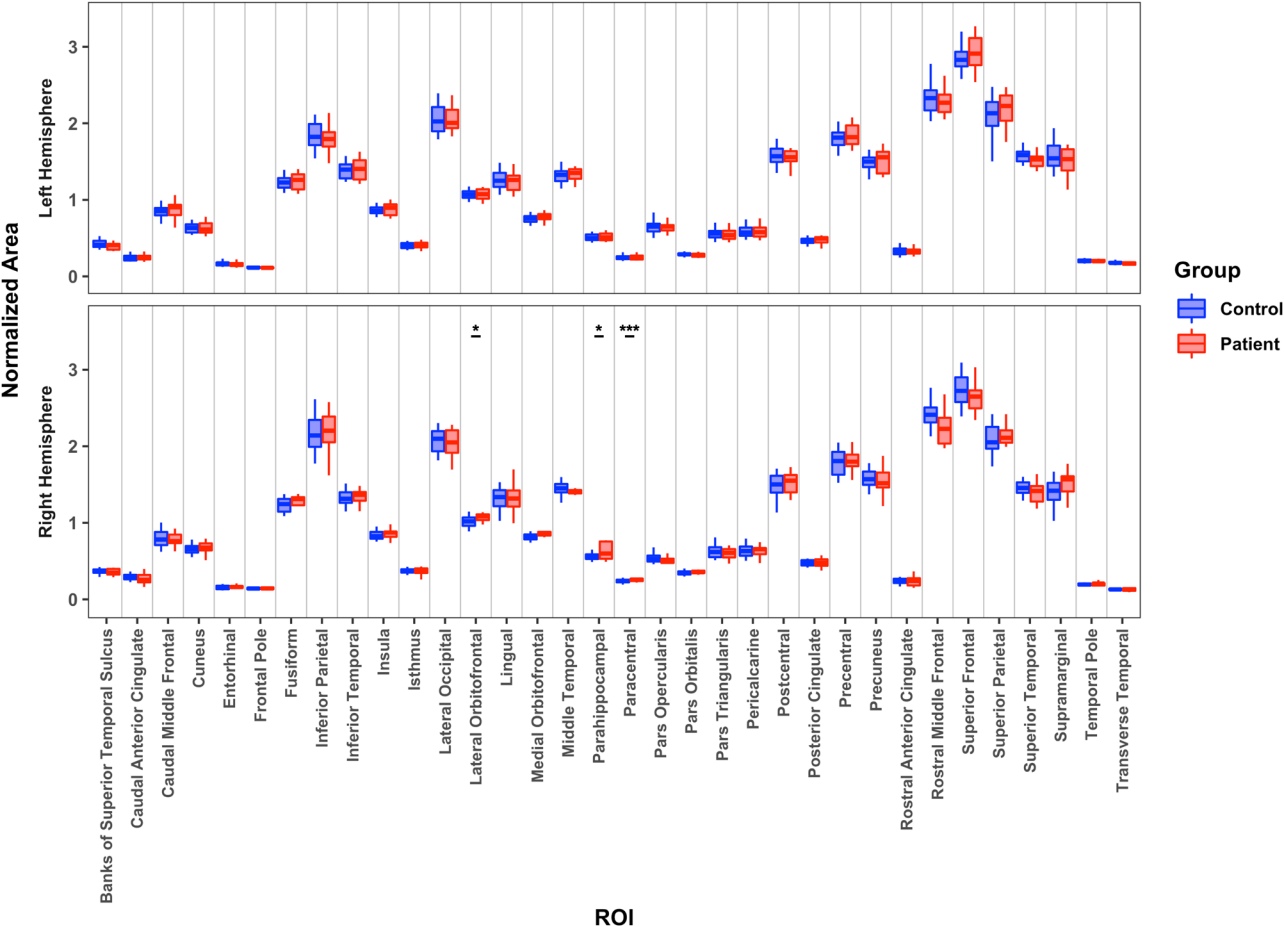
Median normalized cortical surface area for 34 cortical regions for patients’ preserved left hemisphere or right hemisphere relative to controls’ corresponding hemisphere. *: *p* < 0.05; ***: *p* < 0.001.

As depicted in Figure 3, compared with controls’ corresponding hemisphere, patients’ preserved LH did not differ on CSA, whereas patients’ preserved RH had significantly greater CSA in lateral orbitofrontal, paracentral, and parahippocampal cortices. The two patient groups differed from one another with the preserved LH patients having greater CSA in three regions (pars opercularis, rostral anterior cingulate, and transverse temporal) and the preserved RH patients having greater CSA in four regions (frontal pole, inferior parietal, parahippocampal, and pars orbitalis). (See Supplementary Table S5 for descriptive statistics and Supplementary Table S6 for individual *p* and *BF* values.)

In a forward logistic regression analysis with CSA of all 34 areas entered as independent measures for the LH of patients and LH of controls, we could not derive a model associating predictor (region) with outcome (group membership). For the RH patients and controls, the most predictive model with a very strong association value of *r^2^* = 0.83 included the CSA of the entorhinal, lateral orbitofrontal, paracentral, rostral middle frontal, and superior temporal regions. The two patient groups were differentiable by a model containing only the CSA of the pars orbitalis area, with a very strong prediction, *r^2^* = 0.95, and a model with moderate prediction, *r^2^* = 0.33, including only the paracentral area predicted binned ILAE outcome score.

#### 3.2.3 Cortical volume

**Fig. 4.**
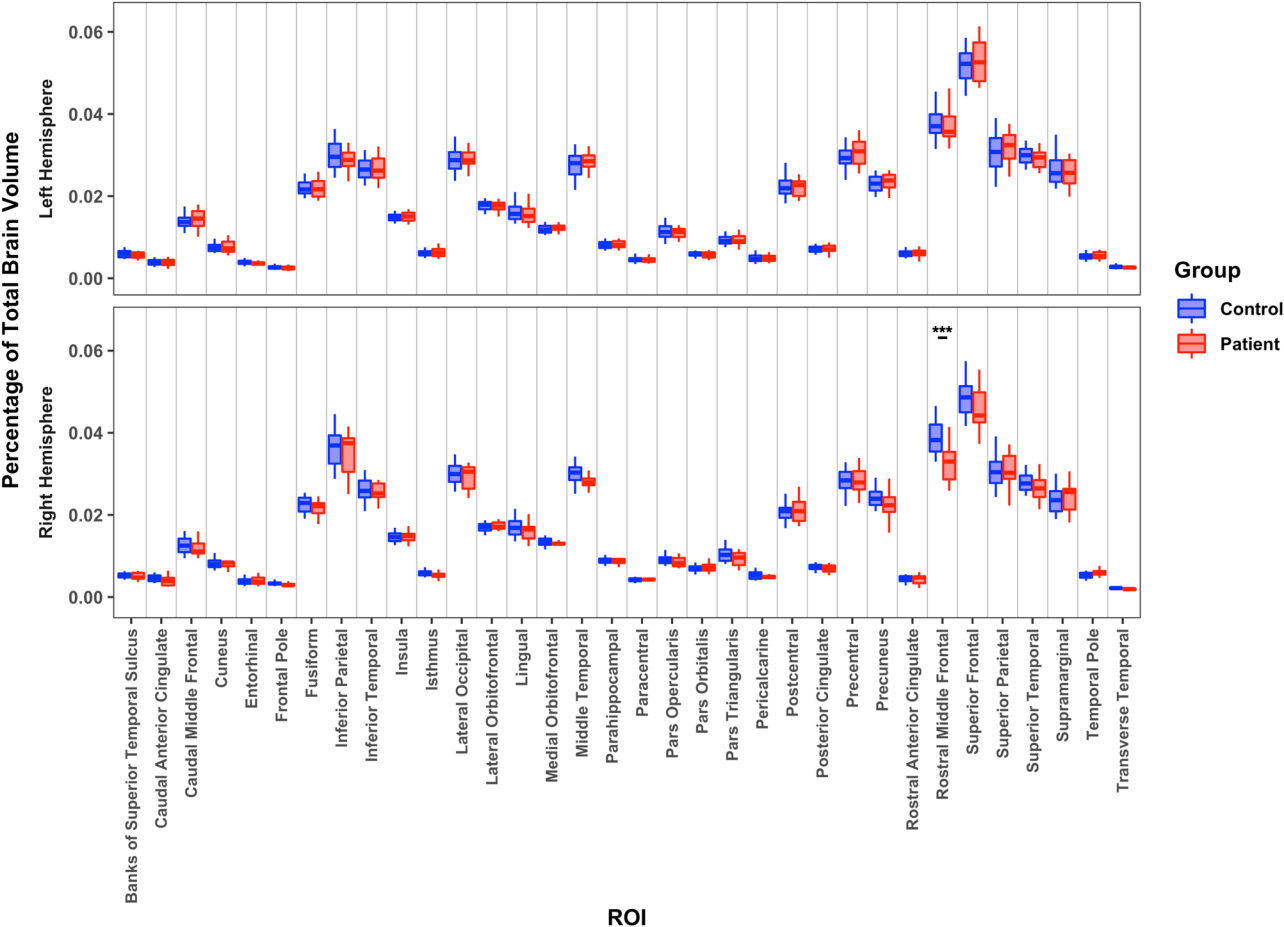
Median region volume (normalized by total volume) for each of 34 cortical regions for patients’ preserved left hemisphere or right hemisphere relative to controls’ corresponding hemisphere. ***: *p* < 0.001.

On the final dependent measure, CV, as shown in Figure 4, the GLM revealed no differences between the LH patients and the LH of the controls. A difference in rostral middle frontal volume was observed between the RH patients and the RH of the controls. A direct comparison between the two patient groups indicated four regions with less volume in the RH than the LH patient group (inferior parietal, pars opercularis, transverse temporal, rostral anterior cingulate) and greater volume in the preserved RH than LH patient group in two regions (pars orbitalis, superior frontal). (See Supplementary Table S7 for descriptive statistics and Supplementary Table S8 for individual *p* and *BF* values.)

A forward logistic regression analysis with the measures of CV for each of the 34 regions predicting the differences between LH patients and controls had no significant predictors. A model with strong predictive value, *r*^2^ = 0.57, which included the rostral middle frontal and pars orbitalis volume predicted group membership for the RH patients vs. RH controls. The RH and LH patients were well differentiated by a model containing pars opercularis and pars orbitalis with very strong predictive value, *r*^2^ = 0.9. Additionally, a model containing CV of paracentral, pars triangularis, and superior parietal volume strongly predicted ILAE score, with *r*^2^ = 0.71.

#### 3.2.4 Summary

**Fig. 5.**
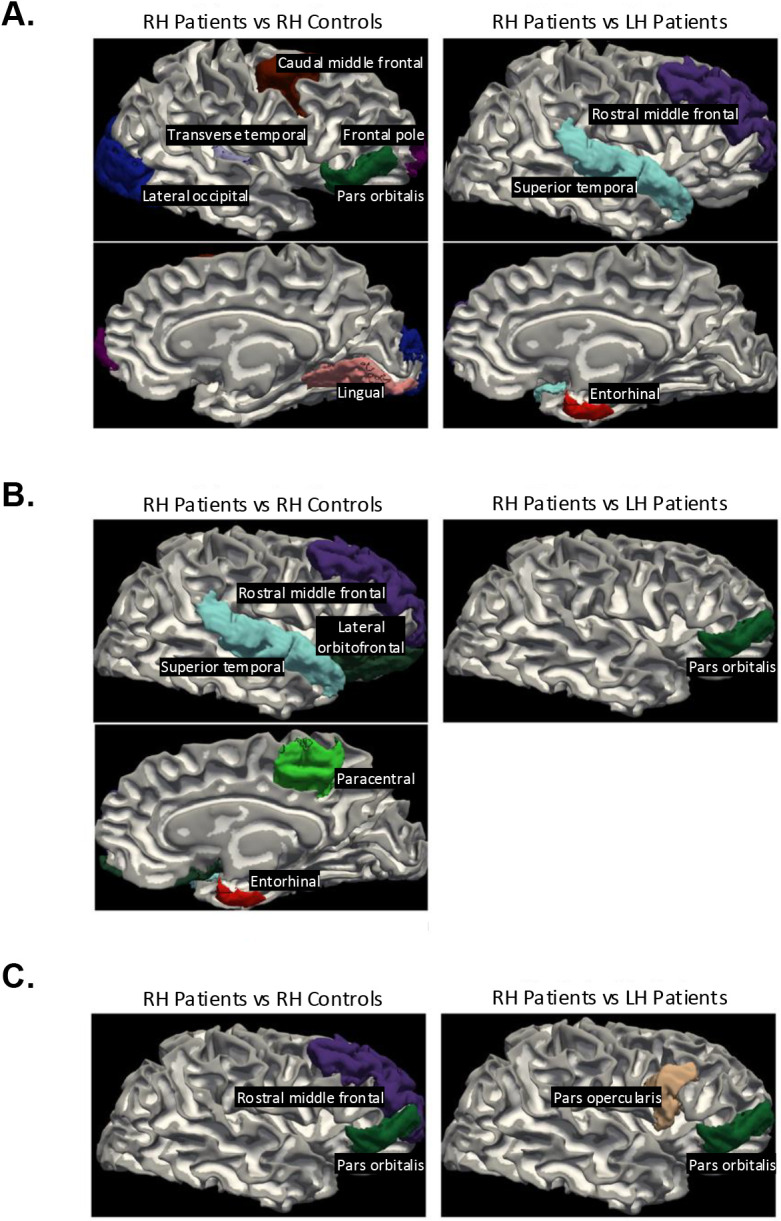
Regions that significantly distinguished between groups (right hemisphere patients versus controls in left columns, right hemisphere patients versus left hemisphere patients in right columns) per binary logistic regression modeling for (A) cortical thickness, (B) cortical surface area, and (C) cortical volume.

In summary, the morphometric differences were more pronounced for the comparison of the RH patient group and the corresponding RH of the controls than for the LH patient/control comparison, and this was true across all three measures and modes of analysis (GLM, bivariate regression). As shown in the lefthand column of Figure 5, the differences in the patients’ preserved RH appear to be largely in language-related regions including pars orbitalis, which borders on Broca’s area, as well as superior temporal and additional frontal regions. We also observed RH patients’ differences relative to controls in homologous language-related regions, in the comparison of the two patient groups against each other, with differences in the superior temporal, pars orbitalis, and pars opercularis (area 44 and part of Broca’s area).

### 3.3 Subcortical regional morphometrics

We segmented (using FreeSurfer) the preserved hemisphere in patients and both hemispheres in controls into nine subcortical regions, namely, the nucleus accumbens (here referred to as accumbens), amygdala, caudate, cerebellum, hippocampus, pallidum, putamen, thalamus, and ventral diencephalon. We extracted the volume of each region.

**Fig. 6.**
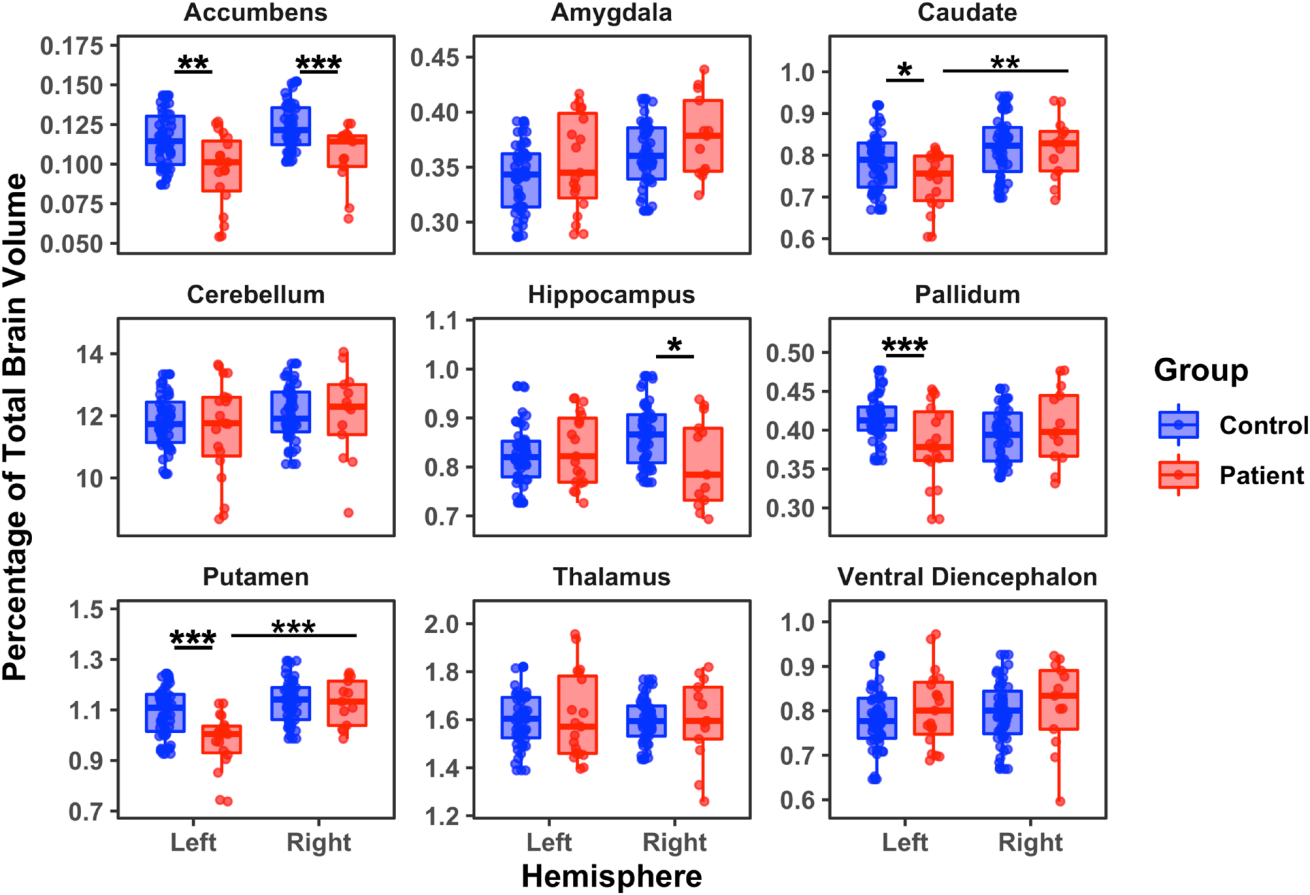
Subcortical regional volumes (normalized) for patients’ preserved left hemisphere or right hemisphere relative to controls’ corresponding hemisphere *: *p* < 0.05; **: *p* < 0.01; ***: *p* < 0.001.

Compared with the LH of controls, patients with a preserved LH showed a significantly lower percentage of whole brain volume of the accumbens, caudate, pallidum, and putamen. Patients with a preserved RH had significantly less volume in the accumbens and hippocampus compared to controls. In the direct contrast between the two patient groups, the LH patient group had significantly less volume in the caudate and putamen relative to the RH patient group (Fig. 6). (See Supplementary Table S9 for descriptive statistics and Supplementary Table S10 for individual *p* and *BF* values.)

To evaluate which of these nine subcortical measures, singly or in combination, best predicted group membership, we used the same forward binary logistic regression procedure as above. The model differentiating LH patients and LH controls yielded a model which included putamen and accumbens, with *r^2^* = 0.44, revealing a strong relationship between these two predictors and group membership. The same analysis for RH patients and RH controls included only the accumbens and yielded an *r^2^* = 0.28, indicating a moderate relationship between this predictor and outcome. A regression model that included the putamen volume alone differentiated strongly, *r^2^* = 0.52, between the preserved RH vs. LH patients. Last, no model including volume of the subcortical measures was reliable in classifying patients into the two ILAE outcome scale bins.

In sum, the GLM results clearly differentiated the LH and RH patients from controls on four and two of the nine subcortical regions, respectively, with the accumbens having lower volume for both patient groups vs. controls. The preserved LH patients had less volume than the RH patients in the putamen and caudate. The regression model identified the accumbens and the putamen as predicting LH patients from controls and the accumbens alone as predictive of group membership in RH patients vs. controls. We could not predict the ILAE outcome scale by any variable/s. These findings indicate that the accumbens and/or putamen are key subcortical structures that differentiate patients from controls, and the putamen plays a vital role in differentiating the side of preserved hemisphere in the patients.

### 3.4 Analysis excluding ablation cases

One possible explanation for the differences between the findings for the LH and RH patients is that the number of cases of resection vs. ablation varies for the two groups. More LH than RH patients have an ablation, which implicates a much smaller region of anatomical change compared to a resection (LH – 7 ablation, 12 resection; RH – 2 ablation, 11 resection). To ensure that the results reported above are not a consequence of this imbalance, we recomputed all the binary logistic regression analyses with only the resection cases (12 LH, 11 RH). Notwithstanding the loss of statistical power with this data reduction, the findings using the three gross measures (LV, GM, and WM) largely mirrored that of the analysis including all patients: whereas no model could predict group membership between LH patients and controls or between LH patients and RH patients, a model with moderate predictive power, *r*^2^ = 0.48, with LV and GM, discriminated between the RH patients and RH controls.

Additionally, the analysis of the cortical regions with only the resection patients revealed largely similar results to that conducted with all the patients. With respect to CxT, a model with weak to moderate predictive value, *r*^2^ = 0.27—which included the fusiform and inferior parietal regions—predicted group membership for LH patients vs. LH controls. We observed a strong association between group membership (patient vs. control) for the RH with the CxT of only two areas, the frontal pole and posterior cingulate, playing a predictive role, *r*^2^ = 0.43. We also obtained a strong prediction of side of preserved hemisphere (LH vs. RH) for separating the two patient groups, *r^2^* = 0.93 based on differences in pars orbitalis CxT. For CSA, no model predicted patients vs. controls for the LH comparisons. However, a model with strong predictive power (including lateral orbitofrontal, parahippocampal, rostral middle frontal, and superior temporal regions), *r*^2^ = 0.76, separated patients and controls in the RH comparisons, and a very strong model of *r*^2^ = 0.90, only with the pars orbitalis, separated the two patient groups. For CV, whereas no model was statistically viable for separating the controls and LH patients, a model with two variables—rostral middle frontal region and pars orbitalis—segregated the controls and RH patients with strong predictive power, *r*^2^ = 0.53. A model with a single variable, pars opercularis, was strongly predictive of whether patients had the LH or RH preserved, *r*^2^ = 0.69. Lastly, no model of the cortical measures was reliable in classifying the LH and RH patients into the two ILAE outcome scale bins.

Finally, the regression analysis using the subcortical regions largely replicated the analysis with all the patients. A model with strong association, *r*^2^ = 0.56, that included the accumbens and putamen separated the LH patients from controls; a reasonably predictive model with *r*^2^ = 0.33 and including the accumbens separated the RH patients from controls, while a model with strong association, *r*^2^ = 0.66, which included only the putamen, separated the two patient groups.

That the findings are almost all replicated with a subset of only those patients who have resections indicates that the results when we included all patients are not an obvious artifact of differences in post-surgical anatomical size of resection/ablation.

### 3.5 Correlations between gross morphometrics and cortical/subcortical morphometrics

In this final analysis, we examined whether any of the gross measures—for example, the size of the LVs—is statistically associated with cortical or subcortical measures. Because the cranium is a fixed size cavity, a large increase in one major feature/tissue such as LV volume will necessarily induce a large decrease in the remaining WM (Lopez et al., 2022). To examine this, we performed bivariate correlations (with multiple comparison correction *p* < 0.0002) between the gross measures and each of the volumes of subcortical regions and then between the gross measures and each of the three dependent measures for each of the 34 cortical parcels. We did this with data from all patients and then separately for LH patients and RH patients. In no analysis did any correlation survive the most stringent multiple comparison, suggesting that group differences in gross anatomy cannot be explained simply by any differences in cortical or subcortical volume.

## 4 Discussion

We undertook a comprehensive characterization of the structural integrity of the post-surgical preserved hemisphere in DRE pediatric patients, including the evaluation of gross anatomy, CxT, CV, and CSA of 34 cortical parcels, as well as of the volume of nine subcortical regions. Studies of post-surgical brain morphometry conducted with adults with DRE have yielded conflicting findings: whereas some have reported reduction of progressive cortical thinning and atrophy, and normalization of cortical thinning rates (Federico & Wiebe, 2020; Galovic et al., 2019, 2020; W. Li et al., 2022; McDonald, 2021), others have noted ongoing, progressive atrophy (and other postsurgical changes (Janson et al., 2024)) even in regions remote from the resection site (Caciagli et al., 2017; Park et al., 2022). Almost all such studies have been carried out in adults—primarily those with temporal lobe epilepsy and anterior temporal lobectomy—and have largely focused on just one variable, such as CxT or CV (but see Zhao et al., 2021 for both).

A clear depiction of the non-resected hemisphere is also important in pediatric cases, however, and is increasingly pressing as the anatomical integrity of the preserved hemisphere is linked with post-surgical behavioral function in this population (Skirrow et al., 2015; Zhao et al., 2021). Furthermore, surgery for those with DRE, especially during childhood, is becoming a first-line form of intervention (Consales et al., 2021; Cross et al., 2022; Vakharia et al., 2018), which can result in cessation of the downward presurgical neuropsychological trajectory (Eriksson et al., 2024). It is also unwise to extrapolate findings from the studies of adult patients to pediatric patients given the greater potential for plasticity in the latter over the former.

Here, we recruited 32 post-surgical pediatric patients, with resections ranging from large removal of tissue, as in cases with hemispherectomy, to more focal alterations such as after robot-assisted laser ablation. On all metrics, we first compared the patients’ preserved hemisphere to the corresponding hemisphere of the controls, and then compared directly the morphometry of the patients with a preserved LH with that of patients with a preserved RH. In addition to the detailed measurement of gross anatomical indices and integrity of cortical and subcortical regions, we also examined differences as a function of which hemisphere was preserved and analyzed correlations across the various metrics. Last, to ensure that the findings were not an artifact of whether the surgical intervention was a resection vs. an ablation, we recomputed all analyses while excluding those patients with laser ablation. All statistical analyses were conducted using both GLM and binary logistic regression analyses, and the results were largely, although not always perfectly, converging. Several key findings emerged from the data (see summary in Table 2).

**Table 2.** At-a-glance summary of all results for univariate and multivariate analyses.

Measures	LH patients/LH controls	RH patients/RH controls	RH patients/LH patients
I. Gross volumes			
*General linear model*			
Lateral Ventricle	patients > controls	patients > controls	n.s.
Gray Matter	n.s.	patients < controls	RH < LH
White Matter	patients < controls	patients < controls	n.s.
Logistic regression	LV and WM	GM and LV	GM
II. Cortical measurements			
*Cortical Thickness*			
General linear model	n.s.	patients < controls: caudal middle frontal, cuneus, frontal pole, fusiform, insula, isthmus, lateral orbitofrontal, lingual, middle temporal, paracentral, posterior cingulate, precuneus, rostral middle frontal, superior frontal, transverse temporal	RH < LH: caudal middle frontal, rostral middle frontal, superior frontal
Logistic regression	n.s.	caudal middle frontal, frontal pole, lateral occipital, lingual, pars orbitalis, transverse temporal	entorhinal, rostral middle frontal, superior temporal
*Cortical surface area*			
General linear model	n.s.	patients > controls: lateral orbitofrontal, parahippocampal, paracentral	LH > RH: pars opercularis, rostral anterior cingulate, transverse temporal; RH > LH: frontal pole, inferior parietal, parahippocampal, pars orbitalis
Logistic regression	superior temporal	entorhinal, lateral orbitofrontal, paracentral, rostral middle frontal, superior temporal	pars orbitalis
*Cortical Volume*			
General linear model	n.s.	patients < controls: rostral middle frontal	LH > RH: pars opercularis, rostral anterior cingulate, superior frontal, transverse temporal; RH > LH: inferior parietal, pars orbitalis
Logistic regression	n.s.	rostral middle frontal, pars orbitalis	pars opercularis, pars orbitalis
III. Subcortical volumes			
General linear model	patients < controls: accumbens, caudate, pallidum, putamen	patients < controls: accumbens, hippocampus	RH > LH: caudate, putamen
Logistic regression	accumbens, putamen	accumbens	putamen

n.s.: not significant.

LH: left hemisphere.

RH: right hemisphere.

First, both LH and RH patient groups differed from controls, having larger LVs and less WM; only RH patients had less GM than controls. The volume of GM alone successfully predicted whether patients had a preserved LH vs. RH, with the former having less atrophy than the latter. In the analysis of the 34 cortical regions, the morphometric measures of the brains of patients with a preserved LH were more like the corresponding hemisphere of controls than was the case for patients with a preserved RH. The preserved RH patients differed from controls on multiple dependent measures in both the GLM analyses and binary logistic regressions. For example, we observed lower CxT in a host of frontal, temporal, and occipital areas and in the isthmus, insula, and precuneus. These patients also had greater CSA than controls in lateral orbitofrontal, parahippocampal, and paracentral areas, and a subset of these areas predicted the group membership (patient vs. control) in the best fit regression model. Lastly, the RH patients had less CV of the rostral middle frontal area (and no obvious parietal region differences) relative to controls, which, together with the pars orbitalis, gave rise to appropriate assignment of membership into patients vs. controls. In sum, whereas the morphometric differences between those with a preserved RH and controls’ RH was striking, those with a preserved LH showed few morphometric differences compared to controls’ LH (only larger ventricles, reduced white matter, select subcortical differences; and no differences in cortical thickness, surface area, or volume).

By inference, unsurprisingly, the direct comparison between the two patient groups also revealed a host of differences based on the side of the preserved hemisphere, with the preserved RH exhibiting lower CxT in frontal, entorhinal, and superior temporal regions, less CSA in frontal cingulate and transverse temporal regions, but also greater CSA in, for example, frontal pole, parahippocampal, and pars orbitalis. Lastly, those with a preserved RH had less CV (more atrophy) in a set of frontal and temporal regions, and, consistent with the CSA measurements, greater CV in the inferior parietal and pars orbitalis regions. Between patient groups, differences in the pars orbitalis and the pars opercularis manifested as key sites in the CSA and CV in both the GLM and regressions analyses.

With respect to the nine subcortical regions, the volume of the putamen and accumbens (along with other structures such as pallidum, caudate, and hippocampus) was lower in both patient groups vs. controls, and the volume of the putamen alone (less volume in LH than RH) was able to differentiate the two patient groups from each other. The indices for the gross measures showed no correlation with any other subcortical or cortical metrics that survived stringent familywise correction, indicating independence between the morphometric changes of LV, WM and GM volume, and other measurements, and confirming the findings that morphometric differences may be more region-specific than global (Sele et al., 2021).

In summary, the key findings we present here include numerous differences in all three dependent measures between patients’ preserved RH and controls’ RH or between patients’ preserved LH and controls’ LH. There are far fewer differences in patients’ preserved LH and controls’ LH. In almost all instances, the preserved RH has reductions in CxT and CV but, surprisingly, has larger CSA in a few regions including orbitofrontal and parahippocampal regions, along with alterations in subcortical regions, namely in the accumbens, caudate, and putamen. These findings are largely consistent with one of the only studies that has examined morphometry following pediatric epilepsy surgery. In that investigation, patients with left but not right hemispherotomy showed pre- to postoperative changes in CV in the preserved hemisphere (mostly decreased volume, but in select regions, increased volume). Patients with left hemispherotomy also had more widespread pre- to post-surgical decreases in CxT of the frontal, parietal, and temporal lobes than those with right hemispherotomy (Zhao et al., 2025). Our findings go beyond this prior work by incorporating a more comprehensive set of morphometric measures (e.g., gross total gray and white matter volumes, CSA, subcortical volumes) in a more heterogeneous patient sample (i.e., not limited to hemispherotomy patients).

### 4.1 Language as a driver of hemispheric plasticity

A key to understanding our findings comes from scrutinizing the regional differences between those with preserved LH vs. RH. Of note, almost all the areas that differ in the preserved RH vs. LH or RH vs. controls are homotopic with or proximal to standard LH language areas, for example, the caudal middle and superior frontal, middle and superior temporal, and transverse temporal regions (see Fig. 5). These findings—derived from an unbiased and whole brain data-driven analysis—appear to converge largely on regions that are implicated in language function. Alterations of LH homologues of language areas in the RH may reflect reorganization of language-dominant cortex, either due to resection or the effects of epilepsy itself. In contrast, a resection of some, most, or all the RH (preserved LH group) results in remarkably few structural/anatomical differences relative to the controls’ LH. Our results are also consistent with one study that revealed that, after left arcuate fasciculus pediatric resection, an increase in the size of the right homotopic region was evident (Goradia et al., 2011). Although other studies have shown widespread changes in structural connectivity in the contralateral hemisphere (e.g., Jeong et al., 2016), our findings have uncovered specificity by hemisphere (only in those with LH resection) and region (largely language-related areas) of post-surgical structural alterations. Our findings attest to the remarkable plasticity and potential for functional reorganization and/or upregulation of language to the RH homotopic frontotemporal regions.

The process of accommodating language function in the RH—which likely has some nascent or “shadow” of language function (Martin et al., 2022; Newport et al., 2022)—potentially underlies the morphometric changes in the RH, especially in childhood (Olulade et al., 2020). Much evidence attests to the fact that, in early childhood, both hemispheres are predisposed to language function (Bates et al., 2001), and even adolescents and young adults who suffer a perinatal ischemic stroke to the LH nevertheless display sentence processing abilities equal to that of non-neurological controls, presumably as a result of RH language engagement (Martin et al., 2022; Newport et al., 2022). The idea of RH plasticity or upregulation for language has also been validated in neuroimaging studies in which, for example, during silent word generation, individuals with peri- or prenatal periventricular LH damage evince RH blood-oxygen-level-dependent activation equal to that observed in the LH of controls (Staudt et al., 2002). We also know that those with language reorganization after left glioma have increased CV in the RH (Pasquini et al., 2022).

What remains unexplained is why we see, relative to controls, a reduction in the morphometric measures of the RH homotopic language regions, rather than their maintenance or even expansion, which is often associated with the assumption of a new function. Presumably, as is also true over the course of typical development, cortical regions are pruned as functions are acquired through the removal of inefficient synapses, dendrites, and neurons (Bourgeois & Rakic, 1996; Changeux & Danchin, 1976; Uddin et al., 2015). Many have suggested that the cortical tissue loss reflects improved neural processing by winnowing brain circuits for select functions (Natu et al., 2019). Cortical thinning is coupled with morphological changes (Natu et al., 2019) and, in childhood, GM thinning specifically is associated with changes in behavior (Sowell et al., 2004), which may be associated with increases in WM (Giedd et al., 1999). Relevantly for this paper, in a longitudinal study, changes of CxT asymmetry in the triangular part of the IFG, which we also identified here, and constituting a portion of Broca’s area in the LH, is suggestive of a neural correlate of language improvement in children’s brains at roughly ages 5–7 years (Qi et al., 2019). Moreover, we should acknowledge that the current investigation only presents structural, not functional, data. Future work should investigate whether morphometric differences in the RH after LH cortical resection are associated with language reorganization, potentially employing a combination of neuropsychological testing and language localizers.

### 4.2 Pre- versus post-surgical changes

One key limitation to the current study is that we only examine patients’ morphometry in the postoperative period. However, it is known that pediatric epilepsy patients who have not undergone surgery show morphometric differences from typically developing children. For example, in one study, pediatric epilepsy patients showed extensive cortical thinning predominantly in frontal and temporal regions (Li et al., 2020), and in another, patients demonstrated cortical thinning in frontal, temporal, and occipital regions (Garcia-Ramos et al., 2015). Machine-learning algorithms have also used structural brain properties to differentiate between epilepsy patients and healthy controls. For example, out of the most commonly used measurements which include CxT, GM volume, WM volume, and cerebrospinal fluid volume, the GM and WM volumes were most informative, accounting for 80% of the group distinctions in the data (Azzony et al., 2023). Azzony et al. (2023) also applied different machine learning classifiers to data extracted from 100 ROIs with K-nearest neighbor approach yielding 97% accuracy in separating control vs. patient group membership (although the authors do not perform inferential statistics to specify the directionality of patient vs. control differences). Our logistic regression procedure, while not always as accurate as that of Azzony et al. (2023), is able to classify our patient vs. control groups, especially for patients’ preserved RH and controls’ RH and did so using only a small number of datapoints (34 for cortical, 9 for subcortical, and 3 for gross anatomy). That there are morphometric differences both in those with focal epilepsy without and with resection suggests that the altered morphometry we document is more likely a consequence of the epileptic activity than an outcome of the surgery per se.

Further support for the presence of pre-surgical morphometric changes comes from studies which compared pre- and post-surgical measures (Govindan et al., 2013; Hertz-Pannier et al., 2002; Zhang et al., 2013). In a recent study on GM changes from pre- to post-surgery (Yu et al., 2024), widespread increases in GM volume were documented in the contralateral cortex and ipsilateral cerebellum. Moreover, Zhao et al. (2025) showed GM changes in superior frontal gyrus and lateral orbitofrontal presurgically, but also a return of prefrontal and cingulate cortices to their normal trajectory post-surgery and persistent post-surgical GM alterations of the temporal lobe. However, both studies sampled only hemispherotomy patients. For a better understanding of morphometric changes resulting specifically from other forms of pediatric epilepsy surgery, future work should examine longitudinal morphometrics of more heterogenous patient samples, like the sample of the current study.

Last, these morphometric differences may be specific to the epilepsy population as they are not obviously observed in other neurological disorders in childhood. Children with traumatic brain injury do not show differences in total brain, WM or GM volumes, or regional subcortical volumes. However, there was some cortical thinning in the angular gyrus and basal forebrain as well as in frontal and occipital regions shortly after injury, but this resolved over several months (Ware et al., 2023).

Notably, our available data sample consisted of primarily postoperative scans, without available postoperative neuropsychological data (e.g., language assessments). Given the cross-sectional, rather than longitudinal design, of the current study, we have been unable to evaluate changes over time and their relationship to behavior. Because of our one-time snapshot, whether the profiles of the childhood DRE brains changed relative to controls’ brains specifically over each the pre-surgical and post-surgical periods and the relationship between these changes over time, hemisphere of resection, and cognitive (specifically language) outcome remain unaddressed and serve as rich fodder for future studies.

### 4.3 Other possible factors that could contribute to differences between LH and RH patients

Because we matched the two groups on age, gender, age at first surgery, seizure-onset age, and ILAE seizure outcome scores, and we included age and gender as covariates in all GLM analyses, none of these factors obviously account for the hemispheric differences in morphometric measures. One further potential difference is that we acquired the data on two different scanners, with arguably different resolution, but this, too, does not obviously explain the asymmetry as both patient groups were roughly equally represented on each scanner (Siemens Verio: 4 RH, 9 LH and Siemens Prisma: 8 RH, 8 LH) and, furthermore, the data were harmonized across magnets. Moreover, due to the patients available at the time of recruitment, by chance, median time from surgery to participation in our study was 3 years for the RH patients and 2 years for the LH patients (and both groups had substantial variability in this metric), and so the interval between age at surgery and age at testing appears not to obviously drive our findings either.

We have also ruled out another potential confound and that concerns a possible difference in the size of the surgery in each group, as we included both ablation and resection cases in this analysis; the re-analysis of the data with only the frank resection cases and no ablation patients, rules out this potential confound. In fact, despite the reduction in statistical power by removing the ablation patients, we replicate almost all the results of the analyses with the data including all the patients. The asymmetry for the preserved LH and RH is also not obviously explained by differences in lobar resections as the LH and RH groups with more focal resections had a roughly equal number of patients with temporal (LH: 4, RH: 4), frontal (LH: 2, RH: 2), occipital (LH: 1, RH: 2), and parietal (LH: 1, RH: 1) resections. Furthermore, an equal number of LH and RH patients had hemispheric surgeries (4 per group), and the majority were disconnection (vs. complete anatomic resection) procedures.

Lastly, one might consider whether participants’ handedness could account for our findings. While we do not have assessments on participants’ preoperative handedness, postoperative handedness is confounded by surgery, as left-sided surgery may affect right motor function and vice versa. In general, however, the association between handedness and differences in cortical morphometry is not clear. For example, in an analysis of more than 30,000 participants, some differences in cortical thickness and surface area between left- and right-handers were noted (Sha et al., 2021), but the regions associated with these differences were distinct from those for which we found patient vs. control differences. Additionally, other studies have suggested that there may be no difference in brain volume or in brain asymmetry related to handedness (Guadalupe et al., 2014; Ocklenburg et al., 2016).

Last, as is clear from our sample, there is much heterogeneity in etiology as well as side and site of resection, and our sample encompasses a wide array of clinical situations associated with DRE which can have different consequences for brain morphology. For example, pre-surgical seizure frequency, especially focal-to-bilateral seizure frequency, is important to consider given that we studied the hemisphere contralateral to the epileptogenic focus. While it is the case that most seizures, if persistent enough, will typically spread to the other hemisphere, this was not the case for our patients. We are confident that in our sample, the patients all had epilepsy lateralized to a single hemisphere. As all were treated with medications prior to resection, any secondary generalization of their focal onset seizures was either controlled or minimized, but surgery for resection was ultimately chosen as a treatment to potentially achieve an Engel 1 outcome to improve quality of life.

### 4.4 Conclusion

Here, we asked, following large pediatric cortical resection, how does the structural integrity of the preserved hemisphere differs in those with large pediatric cortical resection from that of typically developing children? In this case-control study of 32 patients with childhood epilepsy surgery, left-sided—but not right-sided—resection cases showed significantly reduced cortical volume and thickness and increased surface area relative to 51 non-neurological matched controls. There is reorganization of the right hemisphere that is specific to left hemispheric resection but not vice versa, laying the foundation for future work to explore how morphometric changes may be relevant to postoperative remapping of left-lateralized functions (e.g., language) to the right hemisphere.

## Supplementary Material

Supplementary Material

## Data Availability

Relevant data and code are available on Carnegie Mellon University’s Kilthub repository (operated via Figshare): https://doi.org/10.1184/R1/24153423

## References

[IMAG.a.1194-b1] Azzony, S., Moria, K., & Alghamdi, J. (2023). Detecting cortical thickness changes in epileptogenic lesions using machine learning. Brain Sci, 13(3), 487. 10.3390/brainsci13030487 36979297 PMC10046408

[IMAG.a.1194-b2] Bates, E., Reilly, J., Wulfeck, B., Dronkers, N., Opie, M., Fenson, J., Kriz, S., Jeffries, R., Miller, L., & Herbst, K. (2001). Differential effects of unilateral lesions on language production in children and adults. Brain Lang, 79(2), 223–265. 10.1006/brln.2001.2482 11712846

[IMAG.a.1194-b3] Benjamini, Y., & Yekutieli, D. (2001). The control of the false discovery rate in multiple testing under dependency. Ann Statist, 29(4), 1165–1188. https://www.jstor.org/stable/2674075

[IMAG.a.1194-b4] Bourgeois, J. P., & Rakic, P. (1996). Synaptogenesis in the occipital cortex of macaque monkey devoid of retinal input from early embryonic stages. Eur J Neurosci, 8(5), 942–950. 10.1111/j.1460-9568.1996.tb01581.x 8743742

[IMAG.a.1194-b5] Caciagli, L., Bernasconi, A., Wiebe, S., Koepp, M. J., Bernasconi, N., & Bernhardt, B. C. (2017). A meta-analysis on progressive atrophy in intractable temporal lobe epilepsy: Time is brain? Neurology, 89(5), 506–516. 10.1212/WNL.0000000000004176 28687722 PMC5539734

[IMAG.a.1194-b6] Changeux, J. P., & Danchin, A. (1976). Selective stabilisation of developing synapses as a mechanism for the specification of neuronal networks. Nature, 264(5588), 705–712. 10.1038/264705a0 189195

[IMAG.a.1194-b7] Consales, A., Casciato, S., Asioli, S., Barba, C., Caulo, M., Colicchio, G., Cossu, M., de Palma, L., Morano, A., Vatti, G., Villani, F., Zamponi, N., Tassi, L., Di Gennaro, G., & Marras, C. E. (2021). The surgical treatment of epilepsy. Neurol Sci, 42(6), 2249–2260. 10.1007/s10072-021-05198-y 33797619

[IMAG.a.1194-b8] Cross, J. H., Reilly, C., Gutierrez Delicado, E., Smith, M. L., & Malmgren, K. (2022). Epilepsy surgery for children and adolescents: Evidence-based but underused. Lancet Child Adolesc Health, 6(7), 484–494. 10.1016/S2352-4642(22)00098-0 35568054

[IMAG.a.1194-b9] Desikan, R. S., Segonne, F., Fischl, B., Quinn, B. T., Dickerson, B. C., Blacker, D., Buckner, R. L., Dale, A. M., Maguire, R. P., Hyman, B. T., Albert, M. S., & Killiany, R. J. (2006). An automated labeling system for subdividing the human cerebral cortex on MRI scans into gyral based regions of interest. Neuroimage, 31(3), 968–980. 10.1016/j.neuroimage.2006.01.021 16530430

[IMAG.a.1194-b10] Eriksson, M. H., Prentice, F., Piper, R. J., Wagstyl, K., Adler, S., Chari, A., Booth, J., Moeller, F., Das, K., Eltze, C., Cooray, G., Perez Caballero, A., Menzies, L., McTague, A., Shavel-Jessop, S., Tisdall, M. M., Cross, J. H., Martin Sanfilippo, P., & Baldeweg, T. (2024). Long-term neuropsychological trajectories in children with epilepsy: Does surgery halt decline? Brain, 147(8), 2791–2802. 10.1093/brain/awae121 38643018 PMC11292899

[IMAG.a.1194-b11] Fandakova, Y., & Hartley, C. A. (2020). Mechanisms of learning and plasticity in childhood and adolescence. Dev Cogn Neurosci, 42, 100764. 10.1016/j.dcn.2020.100764 32072937 PMC7013153

[IMAG.a.1194-b12] Federico, P., & Wiebe, S. (2020). Is bad brain worse than no brain? Salvaging the cerebral cortex in epilepsy. Brain, 143(11), 3172–3175. 10.1093/brain/awaa330 33278819

[IMAG.a.1194-b13] Fischl, B., & Dale, A. M. (2000). Measuring the thickness of the human cerebral cortex from magnetic resonance images. Proc Natl Acad Sci U S A, 97(20), 11050–11055. 10.1073/pnas.200033797 10984517 PMC27146

[IMAG.a.1194-b14] Fischl, B., Salat, D. H., Busa, E., Albert, M., Dieterich, M., Haselgrove, C., van der Kouwe, A., Killiany, R., Kennedy, D., Klaveness, S., Montillo, A., Makris, N., Rosen, B., & Dale, A. M. (2002). Whole brain segmentation: Automated labeling of neuroanatomical structures in the human brain. Neuron, 33(3), 341–355. https://www.ncbi.nlm.nih.gov/pubmed/11832223 11832223 10.1016/s0896-6273(02)00569-x

[IMAG.a.1194-b15] Fischl, B., Salat, D. H., van der Kouwe, A. J., Makris, N., Segonne, F., Quinn, B. T., & Dale, A. M. (2004). Sequence-independent segmentation of magnetic resonance images. Neuroimage, 23(Suppl. 1), S69–S84. 10.1016/j.neuroimage.2004.07.016 15501102

[IMAG.a.1194-b16] Fischl, B., van der Kouwe, A., Destrieux, C., Halgren, E., Segonne, F., Salat, D. H., Busa, E., Seidman, L. J., Goldstein, J., Kennedy, D., Caviness, V., Makris, N., Rosen, B., & Dale, A. M. (2004). Automatically parcellating the human cerebral cortex. Cereb Cortex, 14(1), 11–22. 10.1093/cercor/bhg087 14654453

[IMAG.a.1194-b17] Fisher, R. S., Cross, J. H., D’Souza, C., French, J. A., Haut, S. R., Higurashi, N., Hirsch, E., Jansen, F. E., Lagae, L., Moshe, S. L., Peltola, J., Roulet Perez, E., Scheffer, I. E., Schulze-Bonhage, A., Somerville, E., Sperling, M., Yacubian, E. M., & Zuberi, S. M. (2017). Instruction manual for the ILAE 2017 operational classification of seizure types. Epilepsia, 58(4), 531–542. 10.1111/epi.13671 28276064

[IMAG.a.1194-b18] Fjell, A. M., Grydeland, H., Krogsrud, S. K., Amlien, I., Rohani, D. A., Ferschmann, L., Storsve, A. B., Tamnes, C. K., Sala-Llonch, R., Due-Tonnessen, P., Bjornerud, A., Solsnes, A. E., Haberg, A. K., Skranes, J., Bartsch, H., Chen, C. H., Thompson, W. K., Panizzon, M. S., Kremen, W. S.,… Walhovd, K. B. (2015). Development and aging of cortical thickness correspond to genetic organization patterns. Proc Natl Acad Sci U S A, 112(50), 15462–15467. 10.1073/pnas.1508831112 26575625 PMC4687601

[IMAG.a.1194-b19] Fonseca, E., Saaria-Estrada, S., Pareto, D., Turon, M., Quinatana, M., E., S., Abraira, L., Tortajada, C., Rovira, A., & Toledo, M. (2023). Relationship between visuoperceptual functions and parietal structural abnormalities in temporal lobe epilepsy. Brain Imaging Behav, 17(1), 35–43. 10.21203/rs.3.rs-1769718/v1 36357555

[IMAG.a.1194-b21] Fortin, J.-P., Cullen, N., Sheline, Y. I., Taylor, W. D., Aselcioglu, I., Cook, P. A., Adams, P., Cooper, C., Fava, M., McGrath, P. J., McInnis, M., Phillips, M. L., Trivedi, M. H., Weissman, M. M., & Shinohara, R. T. (2018). Harmonization of cortical thickness measurements across scanners and sites. Neuroimage, 167, 104–120. 10.1016/j.neuroimage.2017.11.024 29155184 PMC5845848

[IMAG.a.1194-b22] Frangou, S., Modabbernia, A., Williams, S. C. R., Papachristou, E., Doucet, G. E., Agartz, I., Aghajani, M., Akudjedu, T. N., Albajes-Eizagirre, A., Alnaes, D., Alpert, K. I., Andersson, M., Andreasen, N. C., Andreassen, O. A., Asherson, P., Banaschewski, T., Bargallo, N., Baumeister, S., Baur-Streubel, R.,… Dima, D. (2022). Cortical thickness across the lifespan: Data from 17,075 healthy individuals aged 3–90 years. Hum Brain Mapp, 43(1), 431–451. 10.1002/hbm.25364 33595143 PMC8675431

[IMAG.a.1194-b23] Galovic, M., de Tisi, J., McEvoy, A. W., Miserocchi, A., Vos, S. B., Borzi, G., Cueva Rosillo, J., Vuong, K. A., Nachev, P., Duncan, J. S., & Koepp, M. J. (2020). Resective surgery prevents progressive cortical thinning in temporal lobe epilepsy. Brain, 143(11), 3262–3272. 10.1093/brain/awaa284 33179036 PMC7719024

[IMAG.a.1194-b24] Galovic, M., van Dooren, V. Q. H., Postma, T., Vos, S. B., Caciagli, L., Borzi, G., Rosillo, J. C., Vuong, K. A., de Tisi, J., Nachev, P., Duncan, J. S., & Koepp, M. J. (2019). Progressive cortical thinning in patients with focal epilepsy. JAMA Neurol, 76(10), 1230–1239. 10.1001/jamaneurol.2019.1708 31260004 PMC6604082

[IMAG.a.1194-b25] Garcia-Ramos, C., Jackson, D. C., Lin, J. J., Dabbs, K., Jones, J. E., Hsu, D. A., Stafstrom, C. E., Zawadzki, L., Seidenberg, M., Prabhakaran, V., & Hermann, B. P. (2015). Cognition and brain development in children with benign epilepsy with centrotemporal spikes. Epilepsia, 56(10), 1615–1622. 10.1111/epi.13125 26337046 PMC4593750

[IMAG.a.1194-b26] Giedd, J. N., Blumenthal, J., Jeffries, N. O., Castellanos, F. X., Liu, H., Zijdenbos, A., Paus, T., Evans, A. C., & Rapoport, J. L. (1999). Brain development during childhood and adolescence: A longitudinal MRI study. Nat Neurosci, 2(10), 861–863. 10.1038/13158 10491603

[IMAG.a.1194-b27] Glen, D., Levenstein, J. M., Granovetter, M. C., Maallo, A. M. S., & Behrmann, M. (2021). LARGE lesion brain alignment with AFNI. Annual Meeting of the Organization of Human Brain Mapping, 1690. https://afni.nimh.nih.gov/pub/dist/HBM2021/largelesionalignment.pdf

[IMAG.a.1194-b28] Goradia, D., Chugani, H. T., Govindan, R. M., Behen, M., Juhasz, C., & Sood, S. (2011). Reorganization of the right arcuate fasciculus following left arcuate fasciculus resection in children with intractable epilepsy. J Child Neurol, 26(10), 1246–1251. 10.1177/0883073811402689 21551371

[IMAG.a.1194-b29] Govindan, R. M., Brescoll, J., & Chugani, H. T. (2013). Cerebellar pathway changes following cerebral hemispherectomy. J Child Neurol, 28(12), 1548–1554. 10.1177/0883073812455101 22965564

[IMAG.a.1194-b30] Granovetter, M. C., Maallo, A. M. S., Ling, S., Robert, S., Freud, E., Patterson, C., & Behrmann, M. (2024). Functional resilience of the neural visual recognition system post-pediatric occipitotemporal resection. iScience, 27(12), 111440. 10.1016/j.isci.2024.111440 39735436 PMC11681899

[IMAG.a.1194-b31] Grinenko, O., Li, J., Mosher, J. C., Wang, I. Z., Bulacio, J. C., Gonzalez-Martinez, J., Nair, D., Najm, I., Leahy, R. M., & Chauvel, P. (2018). A fingerprint of the epileptogenic zone in human epilepsies. Brain, 141(1), 117–131. 10.1093/brain/awx306 29253102 PMC5837527

[IMAG.a.1194-b32] Guadalupe, T., Willems, R. M., Zwiers, M. P., Arias Vasquez, A., Hoogman, M., Hagoort, P., Fernandez, G., Buitelaar, J., Franke, B., Fisher, S. E., & Francks, C. (2014). Differences in cerebral cortical anatomy of left- and right-handers. Front Psychol, 5, 261. 10.3389/fpsyg.2014.00261 24734025 PMC3975119

[IMAG.a.1194-b33] Han, X., Jovicich, J., Salat, D., van der Kouwe, A., Quinn, B., Czanner, S., Busa, E., Pacheco, J., Albert, M., Killiany, R., Maguire, P., Rosas, D., Makris, N., Dale, A., Dickerson, B., & Fischl, B. (2006). Reliability of MRI-derived measurements of human cerebral cortical thickness: The effects of field strength, scanner upgrade and manufacturer. Neuroimage, 32(1), 180–194. 10.1016/j.neuroimage.2006.02.051 16651008

[IMAG.a.1194-b34] Helmstaedter, C., Beeres, K., Elger, C. E., Kuczaty, S., Schramm, J., & Hoppe, C. (2020). Cognitive outcome of pediatric epilepsy surgery across ages and different types of surgeries: A monocentric 1-year follow-up study in 306 patients of school age. Seizure, 77, 86–92. 10.1016/j.seizure.2019.07.021 31375336

[IMAG.a.1194-b35] Hertz-Pannier, L., Chiron, C., Jambaque, I., Renaux-Kieffer, V., Van de Moortele, P. F., Delalande, O., Fohlen, M., Brunelle, F., & Le Bihan, D. (2002). Late plasticity for language in a child’s non-dominant hemisphere: A pre- and post-surgery fMRI study. Brain, 125(Pt 2), 361–372. 10.1093/brain/awf020 11844736

[IMAG.a.1194-b36] Hubener, M., & Bonhoeffer, T. (2014). Neuronal plasticity: Beyond the critical period. Cell, 159(4), 727–737. 10.1016/j.cell.2014.10.035 25417151

[IMAG.a.1194-b37] Jang, H., Lee, J. Y., Lee, K. I., & Park, K. M. (2017). Are there differences in brain morphology according to handedness? Brain Behav, 7(7), e00730. 10.1002/brb3.730 28729936 PMC5516604

[IMAG.a.1194-b38] Janson, A., Sainburg, L., Akbarian, B., Johnson, G. W., Rogers, B. P., Chang, C., Englot, D. J., & Morgan, V. L. (2024). Indirect structural changes and reduced controllability after temporal lobe epilepsy resection. Epilepsia, 65(3), 675–686. 10.1111/epi.17889 38240699 PMC10948308

[IMAG.a.1194-b39] Jeong, J. W., Asano, E., Juhasz, C., Behen, M. E., & Chugani, H. T. (2016). Postoperative axonal changes in the contralateral hemisphere in children with medically refractory epilepsy: A longitudinal diffusion tensor imaging connectome analysis. Hum Brain Mapp, 37(11), 3946–3956. 10.1002/hbm.23287 27312605 PMC5053859

[IMAG.a.1194-b40] Jobst, B. C., & Cascino, G. D. (2015). Resective epilepsy surgery for drug-resistant focal epilepsy: A review. JAMA, 313(3), 285–293. 10.1001/jama.2014.17426 25602999

[IMAG.a.1194-b41] Lee, M. D., & Wagenmakers, E. J. (2013). Bayesian cognitive modeling: A practical course. Cambridge University Press. 10.1017/cbo9781139087759

[IMAG.a.1194-b42] Li, W., Jiang, Y., Qin, Y., Li, X., Lei, D., Zhang, H., Luo, C., Gong, Q., Zhou, D., & An, D. (2022). Cortical remodeling before and after successful temporal lobe epilepsy surgery. Acta Neurol Scand, 146(2), 144–151. 10.1111/ane.13631 35506500

[IMAG.a.1194-b43] Li, Z., Zhang, J., Wang, F., Yang, Y., Hu, J., Li, Q., Tian, M., Li, T., Huang, B., Liu, H., & Zhang, T. (2020). Surface-based morphometry study of the brain in benign childhood epilepsy with centrotemporal spikes. Ann Transl Med, 8(18), 1150. 10.21037/atm-20-5845 33240999 PMC7576069

[IMAG.a.1194-b44] Lopez, S. M., Aksman, L. M., Oxtoby, N. P., Vos, S. B., Rao, J., Kaestner, E., Alhusaini, S., Alvim, M., Bender, B., Bernasconi, A., Bernasconi, N., Bernhardt, B., Bonilha, L., Caciagli, L., Caldairou, B., Caligiuri, M. E., Calvet, A., Cendes, F., Concha, L.,… Group, E. N.-E. W. (2022). Event-based modeling in temporal lobe epilepsy demonstrates progressive atrophy from cross-sectional data. Epilepsia, 63(8), 2081–2095. 10.1111/epi.17316 35656586 PMC9540015

[IMAG.a.1194-b45] Lyall, A. E., Shi, F., Geng, X., Woolson, S., Li, G., Wang, L., Hamer, R. M., Shen, D., & Gilmore, J. H. (2015). Dynamic development of regional cortical thickness and surface area in early childhood. Cereb Cortex, 25(8), 2204–2212. 10.1093/cercor/bhu027 24591525 PMC4506327

[IMAG.a.1194-b46] Maallo, A. M. S., Granovetter, M. C., Freud, E., Kastner, S., Pinsk, M. A., Patterson, C., & Behrmann, M. (2020). Large-scale resculpting of cortical circuits in children after surgical resection. Sci Rep, 10(1), 21589. 10.1038/s41598-020-78394-z 33299002 PMC7725819

[IMAG.a.1194-b47] Martin, K. C., Seydell-Greenwald, A., Berl, M. M., Gaillard, W. D., Turkeltaub, P. E., & Newport, E. L. (2022). A weak shadow of early life language processing persists in the right hemisphere of the mature brain. Neurobiol Lang (Camb), 3(3), 364–385. 10.1162/nol_a_00069 35686116 PMC9169899

[IMAG.a.1194-b48] McDonald, C. R. (2021). Flattening the curve: Slowing age-accelerated brain atrophy with epilepsy surgery. Epilepsy Curr, 21(3), 159–161. 10.1177/15357597211001066 34867092 PMC8609580

[IMAG.a.1194-b49] Nagelkerke, N. J. D. (1991). A note on a general definition of the coefficient of determination. Biometrika, 78, 691–692. 10.1093/biomet/78.3.691

[IMAG.a.1194-b50] Natu, V. S., Gomez, J., Barnett, M., Jeska, B., Kirilina, E., Jaeger, C., Zhen, Z., Cox, S., Weiner, K. S., Weiskopf, N., & Grill-Spector, K. (2019). Apparent thinning of human visual cortex during childhood is associated with myelination. Proc Natl Acad Sci U S A, 116(41), 20750–20759. 10.1073/pnas.1904931116 31548375 PMC6789966

[IMAG.a.1194-b51] Newport, E. L., Seydell-Greenwald, A., Landau, B., Turkeltaub, P. E., Chambers, C. E., Martin, K. C., Rennert, R., Giannetti, M., Dromerick, A. W., Ichord, R. N., Carpenter, J. L., Berl, M. M., & Gaillard, W. D. (2022). Language and developmental plasticity after perinatal stroke. Proc Natl Acad Sci U S A, 119(42), e2207293119. 10.1073/pnas.2207293119 36215488 PMC9586296

[IMAG.a.1194-b52] Ocklenburg, S., Friedrich, P., Gunturkun, O., & Genc, E. (2016). Voxel-wise grey matter asymmetry analysis in left- and right-handers. Neurosci Lett, 633, 210–214. 10.1016/j.neulet.2016.09.046 27687715

[IMAG.a.1194-b53] Olulade, O. A., Seydell-Greenwald, A., Chambers, C. E., Turkeltaub, P. E., Dromerick, A. W., Berl, M. M., Gaillard, W. D., & Newport, E. L. (2020). The neural basis of language development: Changes in lateralization over age. Proc Natl Acad Sci U S A, 117(38), 23477–23483. 10.1073/pnas.1905590117 32900940 PMC7519388

[IMAG.a.1194-b54] Park, B. Y., Lariviere, S., Rodriguez-Cruces, R., Royer, J., Tavakol, S., Wang, Y., Caciagli, L., Caligiuri, M. E., Gambardella, A., Concha, L., Keller, S. S., Cendes, F., Alvim, M. K. M., Yasuda, C., Bonilha, L., Gleichgerrcht, E., Focke, N. K., Kreilkamp, B. A. K., Domin, M.,… Bernhardt, B. C. (2022). Topographic divergence of atypical cortical asymmetry and atrophy patterns in temporal lobe epilepsy. Brain, 145(4), 1285–1298. 10.1093/brain/awab417 35333312 PMC9128824

[IMAG.a.1194-b55] Pasquini, L., Jenabi, M., Peck, K. K., & Holodny, A. I. (2022). Language reorganization in patients with left-hemispheric gliomas is associated with increased cortical volume in language-related areas and in the default mode network. Cortex, 157, 245–255. 10.1016/j.cortex.2022.09.014 36356409 PMC10201933

[IMAG.a.1194-b56] Qi, T., Schaadt, G., & Friederici, A. D. (2019). Cortical thickness lateralization and its relation to language abilities in children. Dev Cogn Neurosci, 39, 100704. 10.1016/j.dcn.2019.100704 31476670 PMC6892251

[IMAG.a.1194-b57] Reuter, M., Rosas, H. D., & Fischl, B. (2010). Highly accurate inverse consistent registration: A robust approach. Neuroimage, 53(4), 1181–1196. 10.1016/j.neuroimage.2010.07.020 20637289 PMC2946852

[IMAG.a.1194-b58] Reuter, M., Schmansky, N. J., Rosas, H. D., & Fischl, B. (2012). Within-subject template estimation for unbiased longitudinal image analysis. Neuroimage, 61(4), 1402–1418. 10.1016/j.neuroimage.2012.02.084 22430496 PMC3389460

[IMAG.a.1194-b59] Rossini, L., De Santis, D., Mauceri, R. R., Tesoriero, C., Bentivoglio, M., Maderna, E., Maiorana, A., Deleo, F., de Curtis, M., Tringali, G., Cossu, M., Tumminelli, G., Bramerio, M., Spreafico, R., Tassi, L., & Garbelli, R. (2021). Dendritic pathology, spine loss and synaptic reorganization in human cortex from epilepsy patients. Brain, 144(1), 251–265. 10.1093/brain/awaa387 33221837

[IMAG.a.1194-b60] Sainburg, L. E., Englot, D. J., & Morgan, V. L. (2025). The impact of resective epilepsy surgery on the brain network: Evidence from post-surgical imaging. Brain, 148(6), 1866–1875. 10.1093/brain/awaf026 39854170 PMC12129734

[IMAG.a.1194-b61] Sanders, A. F. P., Baum, G. L., Harms, M. P., Kandala, S., Bookheimer, S. Y., Dapretto, M., Somerville, L. H., Thomas, K. M., Van Essen, D. C., Yacoub, E., & Barch, D. M. (2022). Developmental trajectories of cortical thickness by functional brain network: The roles of pubertal timing and socioeconomic status. Dev Cogn Neurosci, 57, 101145. 10.1016/j.dcn.2022.101145 35944340 PMC9386024

[IMAG.a.1194-b62] Scheffer, I. E., Berkovic, S., Capovilla, G., Connolly, M. B., French, J., Guilhoto, L., Hirsch, E., Jain, S., Mathern, G. W., Moshe, S. L., Nordli, D. R., Perucca, E., Tomson, T., Wiebe, S., Zhang, Y. H., & Zuberi, S. M. (2017). ILAE classification of the epilepsies: Position paper of the ILAE Commission for Classification and Terminology. Epilepsia, 58(4), 512–521. 10.1111/epi.13709 28276062 PMC5386840

[IMAG.a.1194-b63] Schnack, H. G., van Haren, N. E., Brouwer, R. M., Evans, A., Durston, S., Boomsma, D. I., Kahn, R. S., & Hulshoff Pol, H. E. (2015). Changes in thickness and surface area of the human cortex and their relationship with intelligence. Cereb Cortex, 25(6), 1608–1617. 10.1093/cercor/bht357 24408955

[IMAG.a.1194-b64] Ségonne, F., Dale, A. M., Busa, E., Glessner, M., Salat, D., Hahn, H. K., & Fischl, B. (2004). A hybrid approach to the skull stripping problem in MRI. Neuroimage, 22(3), 1060–1075. 10.1016/j.neuroimage.2004.03.032 15219578

[IMAG.a.1194-b65] Sele, S., Liem, F., Merillat, S., & Jancke, L. (2021). Age-related decline in the brain: A longitudinal study on inter-individual variability of cortical thickness, area, volume, and cognition. Neuroimage, 240, 118370. 10.1016/j.neuroimage.2021.118370 34245866

[IMAG.a.1194-b66] Sha, Z., Pepe, A., Schijven, D., Carrion-Castillo, A., Roe, J. M., Westerhausen, R., Joliot, M., Fisher, S. E., Crivello, F., & Francks, C. (2021). Handedness and its genetic influences are associated with structural asymmetries of the cerebral cortex in 31,864 individuals. Proc Natl Acad Sci U S A, 118(47), e2113095118. 10.1073/pnas.2113095118 34785596 PMC8617418

[IMAG.a.1194-b67] Sharawat, I. K., Panda, P. K., & Kasinathan, A. (2021). Atypical handedness and its clinicoradiological predictors in children with focal epilepsy. Epilepsy Res, 173, 106622. 10.1016/j.eplepsyres.2021.106622 33813361

[IMAG.a.1194-b68] Skirrow, C., Cross, J. H., Cormack, F., Harkness, W., Vargha-Khadem, F., & Baldeweg, T. (2011). Long-term intellectual outcome after temporal lobe surgery in childhood [Comparative Study Research Support, Non-U.S. Gov’t]. Neurology, 76(15), 1330–1337. 10.1212/WNL.0b013e31821527f0 21482948 PMC3090063

[IMAG.a.1194-b69] Skirrow, C., Cross, J. H., Harrison, S., Cormack, F., Harkness, W., Coleman, R., Meierotto, E., Gaiottino, J., Vargha-Khadem, F., & Baldeweg, T. (2015). Temporal lobe surgery in childhood and neuroanatomical predictors of long-term declarative memory outcome [Research Support, Non-U.S. Gov’t]. Brain, 138(Pt 1), 80–93. 10.1093/brain/awu313 25392199 PMC4285190

[IMAG.a.1194-b70] Sled, J. G., Zijdenbos, A. P., & Evans, A. C. (1998). A nonparametric method for automatic correction of intensity nonuniformity in MRI data. IEEE Trans Med Imaging, 17(1), 87–97. 10.1109/42.668698 9617910

[IMAG.a.1194-b71] Sowell, E. R., Thompson, P. M., Leonard, C. M., Welcome, S. E., Kan, E., & Toga, A. W. (2004). Longitudinal mapping of cortical thickness and brain growth in normal children. J Neurosci, 24(38), 8223–8231. 10.1523/JNEUROSCI.1798-04.2004 15385605 PMC6729679

[IMAG.a.1194-b72] Staudt, M., Lidzba, K., Grodd, W., Wildgruber, D., Erb, M., & Krageloh-Mann, I. (2002). Right-hemispheric organization of language following early left-sided brain lesions: Functional MRI topography. Neuroimage, 16(4), 954–967. 10.1006/nimg.2002.1108 12202083

[IMAG.a.1194-b73] Tang, F., Hartz, A. M. S., & Bauer, B. (2017). Drug-resistant epilepsy: Multiple hypotheses, few answers. Front Neurol, 8, 301. 10.3389/fneur.2017.00301 28729850 PMC5498483

[IMAG.a.1194-b74] TeamCore, R. (2020). R: A Language and Environment for Statistical Computing. R Foundation for Statistical Computing. 10.32614/r.manuals

[IMAG.a.1194-b75] Uddin, L. Q., Supekar, K., Lynch, C. J., Cheng, K. M., Odriozola, P., Barth, M. E., Phillips, J., Feinstein, C., Abrams, D. A., & Menon, V. (2015). Brain state differentiation and behavioral inflexibility in autismdagger. Cereb Cortex, 25(12), 4740–4747. 10.1093/cercor/bhu161 25073720 PMC4635916

[IMAG.a.1194-b76] Vakharia, V. N., Duncan, J. S., Witt, J. A., Elger, C. E., Staba, R., & Engel, J., Jr. (2018). Getting the best outcomes from epilepsy surgery. Ann Neurol, 83(4), 676–690. 10.1002/ana.25205 29534299 PMC5947666

[IMAG.a.1194-b77] Wagenmakers, E. J. (2007). A practical solution to the pervasive problems of p values. Psychon Bull Rev, 14(5), 779–804. 10.3758/bf03194105 18087943

[IMAG.a.1194-b78] Wang, Y., Leiberg, K., Ludwig, T., Little, B., Necus, J. H., Winston, G., Vos, S. B., Tisi, J., Duncan, J. S., Taylor, P. N., & Mota, B. (2021). Independent components of human brain morphology. Neuroimage, 226, 117546. 10.1016/j.neuroimage.2020.117546 33186714 PMC7836233

[IMAG.a.1194-b79] Ware, A. L., Lebel, C., Onicas, A., Abdeen, N., Beauchamp, M. H., Beaulieu, C., Bjornson, B. H., Craig, W., Dehaes, M., Doan, Q., Deschenes, S., Freedman, S. B., Goodyear, B. G., Gravel, J., Ledoux, A. A., Zemek, R., Yeates, K. O., & Pediatric Emergency Research Canada A-CAP Study Group. (2023). Longitudinal gray matter trajectories in pediatric mild traumatic brain injury. Neurology, 101(7), e728–e739. 10.1212/WNL.0000000000207508 37353339 PMC10437012

[IMAG.a.1194-b80] Weil, A. G., Fallah, A., Wang, S., Ibrahim, G. M., Elkaim, L. M., Jayakar, P., Miller, I., Bhatia, S., Niazi, T. N., & Ragheb, J. (2020). Functional hemispherectomy: Can preoperative imaging predict outcome? J Neurosurg Pediatr, 25(6), 567–573. 10.3171/2019.12.PEDS19370 33988937

[IMAG.a.1194-b81] Westman, E., Aguilar, C., Muehlboeck, J. S., & Simmons, A. (2013). Regional magnetic resonance imaging measures for multivariate analysis in Alzheimer’s disease and mild cognitive impairment. Brain Topogr, 26(1), 9–23. 10.1007/s10548-012-0246-x 22890700 PMC3536978

[IMAG.a.1194-b82] Whelan, C. D., Altmann, A., Botia, J. A., Jahanshad, N., Hibar, D. P., Absil, J., Alhusaini, S., Alvim, M. K. M., Auvinen, P., Bartolini, E., Bergo, F. P. G., Bernardes, T., Blackmon, K., Braga, B., Caligiuri, M. E., Calvo, A., Carr, S. J., Chen, J., Chen, S.,… Sisodiya, S. M. (2018). Structural brain abnormalities in the common epilepsies assessed in a worldwide ENIGMA study. Brain, 141(2), 391–408. 10.1093/brain/awx341 29365066 PMC5837616

[IMAG.a.1194-b83] Wierenga, L. M., Langen, M., Oranje, B., & Durston, S. (2014). Unique developmental trajectories of cortical thickness and surface area. Neuroimage, 87, 120–126. 10.1016/j.neuroimage.2013.11.010 24246495

[IMAG.a.1194-b84] Williams, C. M., Peyre, H., & Ramus, F. (2023). Brain volumes, thicknesses, and surface areas as mediators of genetic factors and childhood adversity on intelligence. Cereb Cortex, 33(10), 5885–5895. 10.1093/cercor/bhac468 36533516

[IMAG.a.1194-b85] Winkler, A. M., Kochunov, P., Blangero, J., Almasy, L., Zilles, K., Fox, P. T., Duggirala, R., & Glahn, D. C. (2010). Cortical thickness or grey matter volume? The importance of selecting the phenotype for imaging genetics studies. Neuroimage, 53(3), 1135–1146. 10.1016/j.neuroimage.2009.12.028 20006715 PMC2891595

[IMAG.a.1194-b86] Xue-Ping, W., Hai-Jiao, W., Li-Na, Z., Xu, D., & Ling, L. (2019). Risk factors for drug-resistant epilepsy: A systematic review and meta-analysis. Medicine (Baltimore), 98(30), e16402. 10.1097/MD.0000000000016402 31348240 PMC6708813

[IMAG.a.1194-b87] Yu, H., Chen, Y., Bao, Z., Luo, J., Liu, Q., Qin, P., Wang, C., Qu, J., Wang, W., Cai, L., & Gong, G. (2024). Behavioral and brain morphological changes before and after hemispherotomy. Hum Brain Mapp, 45(13), e70020. 10.1002/hbm.70020 39225128 PMC11369683

[IMAG.a.1194-b88] Zhang, J., Mei, S. S., Liu, Q. Z., Liu, W. F., Chen, H., Xia, H., Zhou, Z., Wang, L., & Li, Y. T. (2013). fMRI and DTI assessment of patients undergoing radical epilepsy surgery. Epilepsy Res, 104(3), 253–263. 10.1016/j.eplepsyres.2012.10.015 23340329 PMC4653737

[IMAG.a.1194-b89] Zhao, Y., Cao, D., Zhu, F., Chen, L., Tan, Z., Chen, T., & Zeng, H. (2025). Structural changes in the gray matter of the contralateral hemisphere and prognosis of motor function in children with pharmacoresistant epilepsy before and after hemispherotomy. Neuroimage, 319, 121429. 10.1016/j.neuroimage.2025.121429 40865623

[IMAG.a.1194-b90] Zhao, Y., Zhang, C., Yang, H., Liu, C., Yu, T., Lu, J., Chen, N., & Li, K. (2021). Recovery of cortical atrophy in patients with temporal lobe epilepsy after successful anterior temporal lobectomy. Epilepsy Behav, 123, 108272. 10.1016/j.yebeh.2021.108272 34500432

