# Supplementary material for: Morphometrics of the preserved post-surgical hemisphere in pediatric drug-resistant epilepsy and implications for post-operative cognition
Source: Imaging Neurosci (Camb). 2026 Apr 6;4:IMAG.a.1194. doi: 10.1162/IMAG.a.1194 (PMC13055013; doi:10.1162/IMAG.a.1194)
Supplement: Supplementary Material [file IMAG.a.1194_supp.pdf]

Table S1 R packages

Package	Version	Use
car[1]	3.0-11	Summarizing statistics
DescTools[2]	0.99.44	Data quality control
lme4[3]	1.1-27.1	Fitting linear mixed models
neuroCombat[4]	1.0.9	Harmonization
psych[5]	2.1.9	Computing descriptive statistics
stringr[6]	1.4.0	Data tidying
tidyverse[7]	1.3.1	Data tidying

Table S2 Descriptive statistics: Median (median absolute deviation) of gross morphometrics

	Control LH	Control RH	Patient LH	Patient RH
LV	5.36x10^3 (1.67x10^3)	4.97x10^3 (1.60x10^3)	6.33x10^3 (3.52x10^3)	5.65x10^3 (3.81x10^3)
GM	2.83x10^5 (2.59x10^4)	2.84x10^5 (2.32x10^4)	2.68x10^5 (3.03x10^4)	2.44x10^5 (2.77x10^4)
WM	2.17x10^5 (2.87x10^4)	2.16x10^5 (2.92x10^4)	2.08x10^5 (2.53x10^4)	1.99x10^5 (2.56x10^4)

LH = left hemisphere
RH = right hemisphere
LV = lateral ventricle
GM = gray matter
WM = white matter

Table S3 Descriptive statistics: Median cortical thickness (Median absolute deviation) per cortical region

Region	Control LH	Control RH	Patient LH	Patient RH
Banks of Superior Temporal Sulcus	2.65 (1.92x10^-1)	2.78 (2.17x10^-1)	2.59 (9.72x10^-2)	2.68 (2.73x10^-1)
Caudal Anterior Cingulate	2.56 (1.98x10^-1)	2.48 (1.54x10^-1)	2.51 (3.12x10^-1)	2.42 (2.45x10^-1)
Caudal Middle Frontal	2.68 (1.57x10^-1)	2.63 (1.38x10^-1)	2.69 (1.35x10^-1)	2.46 (1.92x10^-1)
Cuneus	2.05 (1.46x10^-1)	2.08 (1.72x10^-1)	1.98 (1.38x10^-1)	1.98 (1.33x10^-1)
Entorhinal	3.17 (2.80x10^-1)	3.13 (4.66x10^-1)	3.01 (2.36x10^-1)	3.25 (4.93x10^-1)
Frontal Pole	2.91 (3.22x10^-1)	2.95 (1.96x10^-1)	2.83 (1.76x10^-1)	2.67 (3.05x10^-1)
Fusiform	2.87 (1.02x10^-1)	2.89 (1.07x10^-1)	2.83 (1.46x10^-1)	2.79 (9.55x10^-2)
Inferior Parietal	2.67 (1.43x10^-1)	2.73 (1.63x10^-1)	2.62 (1.25x10^-1)	2.6 (1.00x10^-1)
Inferior Temporal	2.94 (1.34x10^-1)	2.99 (1.61x10^-1)	2.84 (1.42x10^-1)	2.92 (1.38x10^-1)
Insula	3.1 (1.52x10^-1)	3.12 (1.72x10^-1)	3.09 (1.79x10^-1)	3.02 (1.71x10^-1)
Isthmus	2.42 (1.58x10^-1)	2.48 (2.05x10^-1)	2.41 (1.99x10^-1)	2.31 (2.12x10^-1)
Lateral Occipital	2.3 (1.39x10^-1)	2.4 (1.17x10^-1)	2.3 (1.10x10^-1)	2.28 (1.19x10^-1)
Lateral Orbitofrontal	2.75 (1.59x10^-1)	2.74 (1.09x10^-1)	2.74 (1.78x10^-1)	2.67 (1.58x10^-1)
Lingual	2.18 (1.37x10^-1)	2.17 (8.76x10^-2)	2.15 (1.69x10^-1)	2.03 (1.75x10^-1)
Medial Orbitofrontal	2.53 (1.21x10^-1)	2.57 (1.85x10^-1)	2.57 (1.80x10^-1)	2.49 (7.38x10^-2)
Middle Temporal	3.01 (1.43x10^-1)	3.05 (1.39x10^-1)	3.01 (1.24x10^-1)	2.94 (1.99x10^-1)
Parahippocampal	2.64 (1.27x10^-1)	2.59 (2.02x10^-1)	2.64 (1.65x10^-1)	2.57 (1.38x10^-1)
Paracentral	2.85 (2.67x10^-1)	2.8 (1.99x10^-1)	2.78 (3.91x10^-1)	2.59 (2.41x10^-1)
Pars Opercularis	2.8 (1.46x10^-1)	2.77 (1.49x10^-1)	2.68 (1.13x10^-1)	2.59 (2.21x10^-1)
Pars Orbitalis	2.84 (1.95x10^-1)	2.83 (2.64x10^-1)	2.81 (1.37x10^-1)	2.68 (3.19x10^-1)
Pars Triangularis	2.63 (2.12x10^-1)	2.62 (1.58x10^-1)	2.61 (1.83x10^-1)	2.36 (2.35x10^-1)
Pericalcarine	1.73 (1.55x10^-1)	1.75 (1.61x10^-1)	1.73 (9.06x10^-2)	1.64 (1.63x10^-1)
Postcentral	2.27 (1.53x10^-1)	2.22 (1.73x10^-1)	2.26 (1.57x10^-1)	2.23 (3.08x10^-1)
Posterior Cingulate	2.54 (1.32x10^-1)	2.5 (1.79x10^-1)	2.52 (1.62x10^-1)	2.42 (2.08x10^-1)
Precentral	2.72 (1.28x10^-1)	2.65 (1.77x10^-1)	2.71 (1.46x10^-1)	2.56 (1.68x10^-1)
Precuneus	2.62 (1.02x10^-1)	2.63 (1.51x10^-1)	2.58 (1.05x10^-1)	2.51 (1.53x10^-1)
Rostral Anterior Cingulate	2.89 (1.86x10^-1)	2.79 (2.27x10^-1)	2.87 (2.64x10^-1)	2.82 (1.28x10^-1)
Rostral Middle Frontal	2.53 (1.38x10^-1)	2.42 (1.35x10^-1)	2.51 (1.35x10^-1)	2.3 (1.95x10^-1)

Superior Frontal	2.85 (1.70x10^-1)	2.78 (1.57x10^-1)	2.83 (1.41x10^-1)	2.61 (2.19x10^-1)
Superior Parietal	2.41 (1.50x10^-1)	2.4 (1.37x10^-1)	2.38 (1.46x10^-1)	2.33 (1.57x10^-1)
Superior Temporal	3 (1.06x10^-1)	3 (1.60x10^-1)	2.96 (1.24x10^-1)	2.92 (1.65x10^-1)
Supramarginal	2.75 (1.14x10^-1)	2.77 (1.94x10^-1)	2.73 (1.38x10^-1)	2.62 (1.67x10^-1)
Temporal Pole	3.42 (2.00x10^-1)	3.51 (4.05x10^-1)	3.37 (2.97x10^-1)	3.5 (5.69x10^-1)
Transverse Temporal	2.66 (2.07x10^-1)	2.7 (1.95x10^-1)	2.53 (1.49x10^-1)	2.5 (3.54x10^-1)

LH = left hemisphere
RH = right hemisphere

Table S4 General linear model results: Cortical thickness

**Region**	**Comparison**	***p*-value**	***Bayes factor***
Banks of superior temporal sulcus	LC vs. LP	.99	8.33
	RC vs. RP	.15	2.16
	LP vs. RP	.40	2.81
Caudal anterior cingulate	LC vs. LP	.99	8.32
	RC vs. RP	.96	7.99
	LP vs. RP	.29	1.65
Caudal middle frontal	LC vs. LP	.99	8.30
	RC vs. RP	**.02**	4.61x10^-2
	LP vs. RP	**.01**	1.01x10^-2
Cuneus	LC vs. LP	.99	8.34
	RC vs. RP	**.02**	1.05x10^-1
	LP vs. RP	.29	2.24
Entorhinal	LC vs. LP	.99	4.75
	RC vs. RP	.82	7.63
	LP vs. RP	.40	2.84
Frontal pole	LC vs. LP	.99	6.88
	RC vs. RP	**.02**	2.35x10^-2
	LP vs. RP	.18	5.53x10^-1
Fusiform	LC vs. LP	.99	9.48x10^-1
	RC vs. RP	**.02**	4.92x10^-2
	LP vs. RP	.55	4.45
Inferior parietal	LC vs. LP	.99	8.15
	RC vs. RP	.15	1.86
	LP vs. RP	.76	5.36
Inferior temporal	LC vs. LP	.99	2.83
	RC vs. RP	.07	6.62x10^-1
	LP vs. RP	.91	5.61
Insula	LC vs. LP	.99	6.78
	RC vs. RP	**.03**	3.43x10^-1
	LP vs. RP	.40	3.24
Isthmus	LC vs. LP	.99	7.24
	RC vs. RP	**.02**	8.85x10^-2
	LP vs. RP	.22	8.98x10^-1
Lateral occipital	LC vs. LP	.99	7.11
	RC vs. RP	.49	5.31
	LP vs. RP	.52	4.00
Lateral orbitofrontal	LC vs. LP	.99	7.73
	RC vs. RP	**.02**	1.36x10^-1
	LP vs. RP	.22	1.02
Lingual	LC vs. LP	.99	3.23
	RC vs. RP	**.02**	5.65x10^-2
	LP vs. RP	.34	2.20

Medial orbitofrontal	LC vs. LP	.99	8.04
	RC vs. RP	.13	1.65
	LP vs. RP	.18	5.00x10^-1
Middle temporal	LC vs. LP	.99	8.07
	RC vs. RP	**.03**	3.14x10^-1
	LP vs. RP	.40	3.27
Parahippocampal	LC vs. LP	.99	4.50
	RC vs. RP	.06	5.36x10^-1
	LP vs. RP	.29	1.69
Paracentral	LC vs. LP	.99	4.53
	RC vs. RP	**.03**	3.43x10^-1
	LP vs. RP	.26	1.38
Pars opercularis	LC vs. LP	.99	1.39
	RC vs. RP	.06	5.12x10^-1
	LP vs. RP	.19	6.85x10^-1
Pars orbitalis	LC vs. LP	.99	5.07
	RC vs. RP	.95	7.95
	LP vs. RP	.55	4.27
Pars triangularis	LC vs. LP	.99	8.07
	RC vs. RP	.07	8.04x10^-1
	LP vs. RP	.08	1.10x10^-1
Pericalcarine	LC vs. LP	.99	8.08
	RC vs. RP	.10	1.35
	LP vs. RP	.18	2.66x10^-1
Postcentral	LC vs. LP	.99	7.69
	RC vs. RP	.90	7.84
	LP vs. RP	.40	3.01
Posterior cingulate	LC vs. LP	>.99	8.37
	RC vs. RP	**.02**	2.00x10^-1
	LP vs. RP	.18	5.27x10^-1
Precentral	LC vs. LP	.99	5.16
	RC vs. RP	.28	3.19
	LP vs. RP	.22	1.04
Precuneus	LC vs. LP	.99	5.60
	RC vs. RP	**.02**	1.61x10^-1
	LP vs. RP	.18	5.69x10^-1
Rostral anterior cingulate	LC vs. LP	.99	5.12
	RC vs. RP	.69	6.96
	LP vs. RP	.29	1.81
Rostral middle frontal	LC vs. LP	.99	8.20
	RC vs. RP	**.02**	3.75x10^-2
	LP vs. RP	**<.001**	1.06x10^-3
Superior frontal	LC vs. LP	.99	8.13
	RC vs. RP	**.03**	1.84x10^-1
	LP vs. RP	**<.001**	2.65x10^-3
Superior parietal	LC vs. LP	.99	8.15
	RC vs. RP	.14	1.94
	LP vs. RP	.29	1.88
Superior temporal	LC vs. LP	.99	8.05
	RC vs. RP	.28	3.24
	LP vs. RP	.50	3.94
Supramarginal	LC vs. LP	.99	5.66
	RC vs. RP	.07	6.96x10^-1

	LP vs. RP	.22	8.24x10^-1
Temporal pole	LC vs. LP	>.99	8.37
	RC vs. RP	.69	6.90
	LP vs. RP	.40	3.09
Transverse temporal	LC vs. LP	.99	7.45
	RC vs. RP	**.02**	1.50x10^-2
	LP vs. RP	.22	1.08

LC = left hemisphere of controls
LP = left hemisphere of patients
RC = right hemisphere of controls
RP = right hemisphere of patients
p-values less than .05 are bolded.

Table S5 Descriptive statistics: Median normalized cortical surface area (median absolute deviations per cortical area

Region	Control LH	Control RH	Patient LH	Patient RH
Banks of Superior Temporal Sulcus	4.13x10^-1 (5.34x10^-2)	3.65x10^-1 (3.72x10^-2)	4.08x10^-1 (5.47x10^-2)	3.48x10^-1 (5.92x10^-2)
Caudal Anterior Cingulate	2.35x10^-1 (4.14x10^-2)	2.99x10^-1 (4.80x10^-2)	2.39x10^-1 (4.03x10^-2)	2.54x10^-1 (7.73x10^-2)
Caudal Middle Frontal	8.51x10^-1 (7.57x10^-2)	7.82x10^-1 (1.30x10^-1)	9.04x10^-1 (1.24x10^-1)	7.61x10^-1 (1.27x10^-1)
Cuneus	6.34x10^-1 (8.12x10^-2)	6.56x10^-1 (6.47x10^-2)	6.13x10^-1 (6.46x10^-2)	6.75x10^-1 (5.51x10^-2)
Entorhinal	1.70x10^-1 (2.21x10^-2)	1.59x10^-1 (3.39x10^-2)	1.52x10^-1 (3.27x10^-2)	1.62x10^-1 (1.98x10^-2)
Frontal Pole	1.14x10^-1 (1.05x10^-2)	1.42x10^-1 (1.51x10^-2)	1.13x10^-1 (9.08x10^-3)	1.43x10^-1 (2.01x10^-2)
Fusiform	1.23 (9.00x10^-2)	1.24 (1.09x10^-1)	1.26 (1.53x10^-1)	1.31 (8.90x10^-2)
Inferior Parietal	1.82 (1.86x10^-1)	2.14 (2.24x10^-1)	1.79 (1.42x10^-1)	2.2 (2.73x10^-1)
Inferior Temporal	1.39 (1.48x10^-1)	1.31 (1.19x10^-1)	1.4 (1.93x10^-1)	1.37 (9.34x10^-2)
Insula	8.57x10^-1 (5.92x10^-2)	8.27x10^-1 (6.29x10^-2)	8.97x10^-1 (9.83x10^-2)	8.73x10^-1 (6.95x10^-2)
Isthmus	4.10x10^-1 (5.17x10^-2)	3.67x10^-1 (3.49x10^-2)	4.12x10^-1 (6.16x10^-2)	3.76x10^-1 (4.60x10^-2)
Lateral Occipital	2.02 (2.10x10^-1)	2.1 (1.76x10^-1)	2.01 (1.80x10^-1)	2.05 (2.31x10^-1)
Lateral Orbitofrontal	1.07 (6.76x10^-2)	1.02 (8.41x10^-2)	1.07 (9.37x10^-2)	1.09 (7.28x10^-2)
Lingual	1.25 (1.36x10^-1)	1.34 (1.52x10^-1)	1.26 (1.18x10^-1)	1.32 (1.57x10^-1)
Medial Orbitofrontal	7.58x10^-1 (5.75x10^-2)	8.13x10^-1 (4.96x10^-2)	7.75x10^-1 (5.79x10^-2)	8.52x10^-1 (4.46x10^-2)
Middle Temporal	1.33 (8.11x10^-2)	1.45 (8.40x10^-2)	1.35 (7.67x10^-2)	1.41 (4.05x10^-2)
Parahippocampal	5.01x10^-1 (4.98x10^-2)	5.65x10^-1 (4.66x10^-2)	5.09x10^-1 (6.97x10^-2)	5.99x10^-1 (1.05x10^-1)
Paracentral	2.49x10^-1 (2.69x10^-2)	2.37x10^-1 (2.53x10^-2)	2.42x10^-1 (3.98x10^-2)	2.59x10^-1 (2.92x10^-2)
Pars Opercularis	6.51x10^-1 (8.54x10^-2)	5.27x10^-1 (5.57x10^-2)	6.52x10^-1 (5.83x10^-2)	5.06x10^-1 (4.28x10^-2)
Pars Orbitalis	2.88x10^-1 (1.80x10^-2)	3.43x10^-1 (3.24x10^-2)	2.82x10^-1 (2.71x10^-2)	3.55x10^-1 (2.40x10^-2)
Pars Triangularis	5.60x10^-1 (6.30x10^-2)	6.19x10^-1 (9.88x10^-2)	5.40x10^-1 (7.78x10^-2)	6.07x10^-1 (8.17x10^-2)
Pericalcarine	5.73x10^-1 (8.84x10^-2)	6.31x10^-1 (9.16x10^-2)	5.77x10^-1 (9.78x10^-2)	6.56x10^-1 (8.94x10^-2)
Postcentral	1.57 (1.38x10^-1)	1.5 (1.62x10^-1)	1.56 (1.12x10^-1)	1.55 (1.88x10^-1)
Posterior Cingulate	4.64x10^-1 (4.12x10^-2)	4.76x10^-1 (5.24x10^-2)	4.95x10^-1 (5.13x10^-2)	4.78x10^-1 (6.62x10^-2)
Precentral	1.81 (1.18x10^-1)	1.81 (2.23x10^-1)	1.82 (1.57x10^-1)	1.8 (1.28x10^-1)
Precuneus	1.5 (1.06x10^-1)	1.57 (1.40x10^-1)	1.56 (1.32x10^-1)	1.52 (1.08x10^-1)
Rostral Anterior Cingulate	3.23x10^-1 (6.06x10^-2)	2.47x10^-1 (3.67x10^-2)	3.24x10^-1 (4.39x10^-2)	2.42x10^-1 (8.91x10^-2)
Rostral Middle Frontal	2.33 (1.90x10^-1)	2.41 (1.52x10^-1)	2.27 (1.65x10^-1)	2.23 (2.86x10^-1)
Superior Frontal	2.83 (1.49x10^-1)	2.72 (2.39x10^-1)	2.91 (2.96x10^-1)	2.65 (2.26x10^-1)

Superior Parietal	2.13 (2.33x10^-1)	2.05 (2.65x10^-1)	2.23 (2.64x10^-1)	2.11 (1.45x10^-1)
Superior Temporal	1.58 (9.30x10^-2)	1.45 (1.02x10^-1)	1.53 (1.12x10^-1)	1.42 (1.35x10^-1)
Supramarginal	1.54 (1.96x10^-1)	1.42 (1.72x10^-1)	1.53 (2.29x10^-1)	1.57 (1.24x10^-1)
Temporal Pole	2.01x10^-1 (2.81x10^-2)	1.93x10^-1 (1.84x10^-2)	1.97x10^-1 (1.66x10^-2)	2.06x10^-1 (2.80x10^-2)
Transverse Temporal	1.76x10^-1 (1.55x10^-2)	1.30x10^-1 (1.17x10^-2)	1.71x10^-1 (2.63x10^-2)	1.27x10^-1 (2.51x10^-2)

LH = left hemisphere
RH = right hemisphere

Table S6 General linear model results: Cortical surface area

**Region**	**Comparison**	***p*-value**	***Bayes factor***
Banks of superior temporal sulcus	LC vs. LP	.54	0.98
	RC vs. RP	.87	5.79
	LP vs. RP	.10	3.59x10^-1
Caudal anterior cingulate	LC vs. LP	.95	7.93
	RC vs. RP	.29	1.26
	LP vs. RP	.77	4.68
Caudal middle frontal	LC vs. LP	.95	7.31
	RC vs. RP	.91	7.86
	LP vs. RP	.31	1.45
Cuneus	LC vs. LP	.95	8.28
	RC vs. RP	.87	6.16
	LP vs. RP	.39	2.28
Entorhinal	LC vs. LP	.54	1.82
	RC vs. RP	.92	7.96
	LP vs. RP	.97	5.65
Frontal pole	LC vs. LP	.54	1.61
	RC vs. RP	.87	7.44
	LP vs. RP	**<.001**	4.82x10^-6
Fusiform	LC vs. LP	.95	7.72
	RC vs. RP	.87	7.05
	LP vs. RP	.87	5.40
Inferior parietal	LC vs. LP	.81	5.73
	RC vs. RP	.91	7.70
	LP vs. RP	**<.001**	4.62x10^-3
Inferior temporal	LC vs. LP	.86	6.62
	RC vs. RP	.87	7.39
	LP vs. RP	.39	2.30
Insula	LC vs. LP	.86	6.49
	RC vs. RP	.87	5.74
	LP vs. RP	.77	4.53
Isthmus	LC vs. LP	.81	4.85
	RC vs. RP	.87	7.31
	LP vs. RP	.09	2.61x10^-1
Lateral occipital	LC vs. LP	.97	8.36
	RC vs. RP	.87	6.35
	LP vs. RP	.87	5.43
Lateral orbitofrontal	LC vs. LP	.95	8.23
	RC vs. RP	**.02**	4.88x10^-2
	LP vs. RP	.49	2.92
Lingual	LC vs. LP	.95	8.21
	RC vs. RP	.91	7.84
	LP vs. RP	.61	3.54

Medial orbitofrontal	LC vs. LP	.58	2.35
	RC vs. RP	.87	6.54
	LP vs. RP	.19	6.95x10^-1
Middle temporal	LC vs. LP	.81	5.63
	RC vs. RP	.73	4.06
	LP vs. RP	.17	6.31x10^-1
Parahippocampal	LC vs. LP	.95	7.54
	RC vs. RP	**.02**	4.39x10^-2
	LP vs. RP	**<.001**	5.38x10^-3
Paracentral	LC vs. LP	.95	8.30
	RC vs. RP	**<.001**	5.88x10^-2
	LP vs. RP	.39	2.30
Pars opercularis	LC vs. LP	.81	6.17
	RC vs. RP	.59	2.87
	LP vs. RP	**<.001**	2.36x10^-5
Pars orbitalis	LC vs. LP	.58	2.60
	RC vs. RP	.29	1.07
	LP vs. RP	**<.001**	5.96x10^-8
Pars triangularis	LC vs. LP	.95	8.18
	RC vs. RP	.70	3.79
	LP vs. RP	.39	2.22
Pericalcarine	LC vs. LP	.95	8.12
	RC vs. RP	.92	7.92
	LP vs. RP	.39	2.01
Postcentral	LC vs. LP	.81	5.36
	RC vs. RP	.87	5.62
	LP vs. RP	.87	5.34
Posterior cingulate	LC vs. LP	.81	5.35
	RC vs. RP	.87	7.43
	LP vs. RP	.87	5.47
Precentral	LC vs. LP	.81	3.76
	RC vs. RP	.87	5.74
	LP vs. RP	.95	5.61
Precuneus	LC vs. LP	.92	7.08
	RC vs. RP	.87	5.54
	LP vs. RP	.87	5.22
Rostral anterior cingulate	LC vs. LP	.81	5.86
	RC vs. RP	.87	7.58
	LP vs. RP	**<.001**	5.14x10^-3
Rostral middle frontal	LC vs. LP	.95	8.06
	RC vs. RP	.14	3.36
	LP vs. RP	.87	5.10
Superior frontal	LC vs. LP	.81	3.89
	RC vs. RP	.87	7.51
	LP vs. RP	.12	4.17x10^-1
Superior parietal	LC vs. LP	.81	4.69
	RC vs. RP	.91	7.82
	LP vs. RP	.69	3.83
Superior temporal	LC vs. LP	.20	8.12x10^-2
	RC vs. RP	.14	5.88x10^-1
	LP vs. RP	.07	1.51x10^-1
Supramarginal	LC vs. LP	.54	1.78
	RC vs. RP	.14	4.18x10^-1
	LP vs. RP	.87	5.28

Temporal pole	LC vs. LP	.97	8.34
	RC vs. RP	.59	2.95
	LP vs. RP	.87	5.47
Transverse temporal	LC vs. LP	.20	4.64x10^-1
	RC vs. RP	.87	6.76
	LP vs. RP	**<.001**	1.42x10^-5

LC = left hemisphere of controls
LP = left hemisphere of patients
RC = right hemisphere of controls
RP = right hemisphere of patients
p-values less than .05 are bolded.

Table S7 Descriptive statistics: Median normalized region volume (median absolute deviations) per cortical area

Region	Control LH	Control RH	Patient LH	Patient RH
Banks of Superior Temporal Sulcus	5.96x10^-3 (1.10x10^-3)	5.25x10^-3 (7.56x10^-4)	5.58x10^-3 (9.94x10^-4)	4.74x10^-3 (6.36x10^-4)
Caudal Anterior Cingulate	3.71x10^-3 (6.67x10^-4)	4.62x10^-3 (1.01x10^-3)	3.82x10^-3 (9.24x10^-4)	3.89x10^-3 (1.58x10^-3)
Caudal Middle Frontal	1.37x10^-2 (1.50x10^-3)	1.25x10^-2 (2.38x10^-3)	1.45x10^-2 (2.77x10^-3)	1.11x10^-2 (1.37x10^-3)
Cuneus	7.40x10^-3 (1.16x10^-3)	8.04x10^-3 (1.23x10^-3)	7.25x10^-3 (2.17x10^-3)	8.40x10^-3 (6.12x10^-4)
Entorhinal	3.86x10^-3 (5.96x10^-4)	3.71x10^-3 (8.79x10^-4)	3.58x10^-3 (4.69x10^-4)	3.73x10^-3 (1.36x10^-3)
Frontal Pole	2.63x10^-3 (4.52x10^-4)	3.27x10^-3 (4.02x10^-4)	2.44x10^-3 (5.64x10^-4)	2.86x10^-3 (5.00x10^-4)
Fusiform	2.16x10^-2 (1.62x10^-3)	2.29x10^-2 (2.45x10^-3)	2.17x10^-2 (2.95x10^-3)	2.22x10^-2 (2.59x10^-3)
Inferior Parietal	2.96x10^-2 (4.09x10^-3)	3.69x10^-2 (5.81x10^-3)	2.88x10^-2 (3.15x10^-3)	3.75x10^-2 (3.96x10^-3)
Inferior Temporal	2.65x10^-2 (2.97x10^-3)	2.59x10^-2 (3.32x10^-3)	2.62x10^-2 (3.59x10^-3)	2.53x10^-2 (3.01x10^-3)
Insula	1.48x10^-2 (1.03x10^-3)	1.46x10^-2 (1.44x10^-3)	1.50x10^-2 (1.47x10^-3)	1.49x10^-2 (1.52x10^-3)
Isthmus	6.24x10^-3 (6.96x10^-4)	5.76x10^-3 (7.47x10^-4)	6.25x10^-3 (1.41x10^-3)	5.23x10^-3 (6.95x10^-4)
Lateral Occipital	2.87x10^-2 (3.19x10^-3)	3.00x10^-2 (2.83x10^-3)	2.87x10^-2 (2.67x10^-3)	3.05x10^-2 (2.36x10^-3)
Lateral Orbitofrontal	1.79x10^-2 (1.37x10^-3)	1.71x10^-2 (1.23x10^-3)	1.77x10^-2 (1.15x10^-3)	1.71x10^-2 (9.44x10^-4)
Lingual	1.56x10^-2 (2.00x10^-3)	1.68x10^-2 (2.51x10^-3)	1.51x10^-2 (2.95x10^-3)	1.65x10^-2 (2.09x10^-3)
Medial Orbitofrontal	1.18x10^-2 (1.15x10^-3)	1.35x10^-2 (1.18x10^-3)	1.26x10^-2 (8.73x10^-4)	1.30x10^-2 (3.87x10^-4)
Middle Temporal	2.80x10^-2 (3.68x10^-3)	3.03x10^-2 (2.20x10^-3)	2.85x10^-2 (2.30x10^-3)	2.79x10^-2 (1.24x10^-3)
Parahippocampal	8.17x10^-3 (1.07x10^-3)	8.87x10^-3 (7.65x10^-4)	8.09x10^-3 (1.08x10^-3)	8.78x10^-3 (7.56x10^-4)
Paracentral	4.42x10^-3 (6.49x10^-4)	4.22x10^-3 (5.31x10^-4)	4.31x10^-3 (7.23x10^-4)	4.25x10^-3 (2.40x10^-4)
Pars Opercularis	1.12x10^-2 (1.70x10^-3)	8.97x10^-3 (1.05x10^-3)	1.13x10^-2 (1.43x10^-3)	8.28x10^-3 (1.41x10^-3)
Pars Orbitalis	6.00x10^-3 (5.57x10^-4)	6.95x10^-3 (7.75x10^-4)	5.70x10^-3 (1.08x10^-3)	7.09x10^-3 (1.20x10^-3)
Pars Triangularis	9.05x10^-3 (1.24x10^-3)	1.03x10^-2 (2.11x10^-3)	8.96x10^-3 (1.28x10^-3)	9.61x10^-3 (2.15x10^-3)
Pericalcarine	4.67x10^-3 (1.03x10^-3)	5.30x10^-3 (1.17x10^-3)	4.88x10^-3 (1.05x10^-3)	4.92x10^-3 (4.81x10^-4)
Postcentral	2.19x10^-2 (2.52x10^-3)	2.09x10^-2 (1.88x10^-3)	2.27x10^-2 (3.05x10^-3)	2.09x10^-2 (3.52x10^-3)
Posterior Cingulate	7.02x10^-3 (9.60x10^-4)	7.39x10^-3 (7.71x10^-4)	7.04x10^-3 (7.81x10^-4)	6.91x10^-3 (1.18x10^-3)
Precentral	2.93x10^-2 (2.32x10^-3)	2.84x10^-2 (2.95x10^-3)	3.09x10^-2 (3.63x10^-3)	2.79x10^-2 (3.95x10^-3)
Precuneus	2.31x10^-2 (2.53x10^-3)	2.40x10^-2 (2.50x10^-3)	2.38x10^-2 (2.27x10^-3)	2.24x10^-2 (2.60x10^-3)
Rostral Anterior Cingulate	5.94x10^-3 (8.95x10^-4)	4.45x10^-3 (9.42x10^-4)	5.93x10^-3 (8.32x10^-4)	4.79x10^-3 (3.52x10^-4)
Rostral Middle Frontal	3.70x10^-2 (3.57x10^-3)	3.82x10^-2 (4.22x10^-3)	3.57x10^-2 (3.66x10^-3)	3.30x10^-2 (6.44x10^-3)

Superior Frontal	5.22x10^-2 (4.79x10^-3)	4.86x10^-2 (5.25x10^-3)	5.26x10^-2 (7.14x10^-3)	4.42x10^-2 (3.19x10^-3)
Superior Parietal	3.08x10^-2 (5.14x10^-3)	3.04x10^-2 (3.92x10^-3)	3.24x10^-2 (4.33x10^-3)	3.02x10^-2 (2.96x10^-3)
Superior Temporal	3.00x10^-2 (2.53x10^-3)	2.77x10^-2 (2.78x10^-3)	2.94x10^-2 (2.56x10^-3)	2.65x10^-2 (3.12x10^-3)
Supramarginal	2.56x10^-2 (3.74x10^-3)	2.36x10^-2 (3.49x10^-3)	2.57x10^-2 (4.49x10^-3)	2.56x10^-2 (4.01x10^-3)
Temporal Pole	5.23x10^-3 (7.17x10^-4)	5.21x10^-3 (8.66x10^-4)	5.41x10^-3 (1.06x10^-3)	5.88x10^-3 (6.90x10^-4)
Transverse Temporal	2.80x10^-3 (4.28x10^-4)	2.17x10^-3 (2.24x10^-4)	2.61x10^-3 (2.42x10^-4)	1.93x10^-3 (3.26x10^-4)

LH = left hemisphere
RH = right hemisphere

Table S8 General linear model results: Cortical volume

**Region**	**Comparison**	***p*-value**	***Bayes factor***
Banks of superior temporal sulcus	LC vs. LP	.75	2.61
	RC vs. RP	.61	5.78
	LP vs. RP	.27	1.30
Caudal anterior cingulate	LC vs. LP	.99	8.36
	RC vs. RP	.30	1.64
	LP vs. RP	.84	4.97
Caudal middle frontal	LC vs. LP	.75	5.36
	RC vs. RP	.61	5.66
	LP vs. RP	.07	1.66x10^-1
Cuneus	LC vs. LP	.75	5.67
	RC vs. RP	.84	7.57
	LP vs. RP	.84	4.61
Entorhinal	LC vs. LP	.75	1.16
	RC vs. RP	.85	7.67
	LP vs. RP	.45	2.12
Frontal pole	LC vs. LP	.75	2.81
	RC vs. RP	.30	5.04x10^-1
	LP vs. RP	.07	1.92x10^-1
Fusiform	LC vs. LP	.99	8.33
	RC vs. RP	.53	4.70
	LP vs. RP	.97	5.63
Inferior parietal	LC vs. LP	.75	5.72
	RC vs. RP	.79	7.07
	LP vs. RP	**.01**	2.29x10^-3
Inferior temporal	LC vs. LP	.99	7.77
	RC vs. RP	.79	7.24
	LP vs. RP	.80	3.54
Insula	LC vs. LP	.99	7.51
	RC vs. RP	.83	7.45
	LP vs. RP	.97	5.65
Isthmus	LC vs. LP	.75	5.40
	RC vs. RP	.30	7.72x10^-1
	LP vs. RP	.07	1.83x10^-1
Lateral occipital	LC vs. LP	.99	8.36
	RC vs. RP	.73	6.53
	LP vs. RP	.84	4.72
Lateral orbitofrontal	LC vs. LP	.99	7.39
	RC vs. RP	.30	1.27
	LP vs. RP	.83	4.07
Lingual	LC vs. LP	.77	6.25

	RC vs. RP	.45	3.81
	LP vs. RP	.93	5.37
Medial orbitofrontal	LC vs. LP	.75	4.39
	RC vs. RP	.61	5.56
	LP vs. RP	.12	6.06x10^-1
Middle temporal	LC vs. LP	.75	3.14
	RC vs. RP	.30	1.36
	LP vs. RP	.97	5.64
Parahippocampal	LC vs. LP	.99	8.36
	RC vs. RP	.34	2.77
	LP vs. RP	.07	2.39x10^-1
Paracentral	LC vs. LP	.99	8.34
	RC vs. RP	.34	2.85
	LP vs. RP	.93	5.42
Pars opercularis	LC vs. LP	.75	5.70
	RC vs. RP	.30	2.16
	LP vs. RP	**<.001**	3.55x10^-5
Pars orbitalis	LC vs. LP	.75	3.40
	RC vs. RP	.30	2.43
	LP vs. RP	**<.001**	2.34x10^-4
Pars triangularis	LC vs. LP	.99	8.36
	RC vs. RP	.30	2.05
	LP vs. RP	.93	5.51
Pericalcarine	LC vs. LP	.99	8.36
	RC vs. RP	.54	5.04
	LP vs. RP	.97	5.56
Postcentral	LC vs. LP	.99	7.80
	RC vs. RP	.52	4.49
	LP vs. RP	.84	4.99
Posterior cingulate	LC vs. LP	.99	7.80
	RC vs. RP	.30	1.49
	LP vs. RP	.81	3.80
Precentral	LC vs. LP	.75	2.11
	RC vs. RP	.98	7.99
	LP vs. RP	.26	1.04
Precuneus	LC vs. LP	.77	6.09
	RC vs. RP	.30	1.56
	LP vs. RP	.84	4.48
Rostral anterior cingulate	LC vs. LP	.99	8.37
	RC vs. RP	.98	8.00
	LP vs. RP	**<.001**	5.80x10^-3
Rostral middle frontal	LC vs. LP	.99	8.33
	RC vs. RP	**<.001**	1.49x10^-3
	LP vs. RP	.09	3.07x10^-1
Superior frontal	LC vs. LP	.75	3.52
	RC vs. RP	.30	2.08
	LP vs. RP	**.01**	1.39x10^-2
Superior parietal	LC vs. LP	.75	4.66
	RC vs. RP	.88	7.81
	LP vs. RP	.84	4.20
Superior temporal	LC vs. LP	.75	2.56
	RC vs. RP	.30	1.76
	LP vs. RP	.07	2.31x10^-1
Supramarginal	LC vs. LP	.75	5.50

	RC vs. RP	.30	2.29
	LP vs. RP	.84	4.91
Temporal pole	LC vs. LP	.75	5.20
	RC vs. RP	.30	1.61
	LP vs. RP	.87	5.13
Transverse temporal	LC vs. LP	.75	2.39
	RC vs. RP	.30	6.01x10^-1
	LP vs. RP	**<.001**	1.30x10^-4

LC = left hemisphere of controls
LP = left hemisphere of patients
RC = right hemisphere of controls
RP = right hemisphere of patients
p-values less than .05 are bolded.

Table S9 Descriptive statistics: Median volume (median absolute deviations) per subcortical area

Region	Control LH	Control RH	Patient LH	Patient RH
Accumbens	1.14x10^-3 (2.33x10^-4)	1.22x10^-3 (1.97x10^-4)	1.01x10^-3 (2.33x10^-4)	1.14x10^-3 (1.65x10^-4)
Amygdala	3.43x10^-3 (3.95x10^-4)	3.60x10^-3 (3.29x10^-4)	3.45x10^-3 (5.90x10^-4)	3.79x10^-3 (4.80x10^-4)
Caudate	7.89x10^-3 (7.50x10^-4)	8.23x10^-3 (6.95x10^-4)	7.56x10^-3 (6.59x10^-4)	8.29x10^-3 (6.34x10^-4)
Cerebellum	1.17x10^-1 (9.48x10^-3)	1.19x10^-1 (8.94x10^-3)	1.18x10^-1 (1.35x10^-2)	1.23x10^-1 (1.19x10^-2)
Hippocampus	8.20x10^-3 (5.78x10^-4)	8.66x10^-3 (7.55x10^-4)	8.22x10^-3 (9.93x10^-4)	7.84x10^-3 (1.17x10^-3)
Pallidum	4.13x10^-3 (2.32x10^-4)	3.94x10^-3 (4.67x10^-4)	3.78x10^-3 (6.03x10^-4)	3.98x10^-3 (6.22x10^-4)
Putamen	1.11x10^-2 (1.11x10^-3)	1.14x10^-2 (9.41x10^-4)	1.00x10^-2 (9.84x10^-4)	1.13x10^-2 (1.40x10^-3)
Thalamus	1.60x10^-2 (1.26x10^-3)	1.59x10^-2 (9.39x10^-4)	1.57x10^-2 (1.76x10^-3)	1.60x10^-2 (1.81x10^-3)
Ventral Diencephalon	7.77x10^-3 (6.66x10^-4)	8.01x10^-3 (7.78x10^-4)	8.01x10^-3 (9.78x10^-4)	8.34x10^-3 (1.03x10^-3)

LH = left hemisphere
RH = right hemisphere

Table S10 General linear model results: Subcortical volume

Region	Comparison	*p*-value	*Bayes factor*
Accumbens	LC vs. LP	**.01**	1.37x10^-2
	RC vs. RP	**<.001**	1.84x10^-2
	LP vs. RP	.62	3.25
Amygdala	LC vs. LP	.38	3.90
	RC vs. RP	.52	2.99
	LP vs. RP	.22	1.15
Caudate	LC vs. LP	**.02**	1.79x10^-1
	RC vs. RP	.99	7.99
	LP vs. RP	**<.01**	3.28x10^-2
Cerebellum	LC vs. LP	.56	6.31
	RC vs. RP	.93	7.59
	LP vs. RP	.66	4.34
Hippocampus	LC vs. LP	.77	7.80
	RC vs. RP	**.01**	4.75x10^-2
	LP vs. RP	.62	3.84
Pallidum	LC vs. LP	**<.001**	9.38x10^-3
	RC vs. RP	.93	6.07
	LP vs. RP	.22	1.06
Putamen	LC vs. LP	**<.001**	6.32x10^-4
	RC vs. RP	.93	6.99
	LP vs. RP	**<.01**	1.49x10^-3

Thalamus	LC vs. LP	.99	8.37
	RC vs. RP	.93	7.37
	LP vs. RP	.97	5.65
Ventral diencephalon	LC vs. LP	.55	5.39
	RC vs. RP	.99	8.00
	LP vs. RP	.91	5.43

LC = left hemisphere of controls
LP = left hemisphere of patients
RC = right hemisphere of controls
RP = right hemisphere of patients
p-values less than .05 are bolded.

Supplementary Material References

1. Fox J, Weisberg S. *An {R} Companion to Applied Regression*. 3rd ed. Thousand Oaks, CA: Sage; 2019.

2. Signorell A. *DescTools: Tools for Descriptive Statistics*.; 2021.

3. Bates D, Mächler M, Bolker B, Walker S. Fitting linear mixed-effects models using lme4. *J Stat Softw*. 2015;67(1):1-48. doi:10.18637/jss.v067.i01

4. Fortin J-P. *NeuroCombat: Harmonization of Multi-Site Imaging Data with ComBat*.; 2021.

5. Revelle W. *Psych: Procedures for Personality and Psychological Research*. Evanston, Illinois, USA: Northwestern Unviersity; 2021.

6. Wickham H. *Stringr: Simple, Consistent Wrappers for Common String Operations*.; 2019.

7. Wickham H, Averick M, Bryan J, et al. Welcome to the tidyverse. *JOSS*. 2019;4(43):1686. doi:10.21105/joss.01686
